# Distribution and Evolution of Nonribosomal Peptide Synthetase Gene Clusters in the *Ceratocystidaceae*

**DOI:** 10.3390/genes10050328

**Published:** 2019-04-30

**Authors:** Mohammad Sayari, Magriet A. van der Nest, Emma T. Steenkamp, Nicole C. Soal, P. Markus Wilken, Brenda D. Wingfield

**Affiliations:** Department of Biochemistry, Genetics and Microbiology, Forestry and Agricultural Biotechnology Institute, University of Pretoria, Pretoria 0002, South Africa; mohammad.sayari@fabi.up.ac.za (M.S.); magriet.vandernest@fabi.up.ac.za (M.A.v.d.N.); emma.steenkamp@fabi.up.ac.za (E.T.S.); nicole.soal@fabi.up.ac.za (N.C.S.); markus.wilken@fabi.up.ac.za (P.M.W.)

**Keywords:** nonribosomal peptide production, nonribosomal peptide synthetase (NRPS) gene, *Ceratocystidaceae*, siderophore production

## Abstract

In filamentous fungi, genes in secondary metabolite biosynthetic pathways are generally clustered. In the case of those pathways involved in nonribosomal peptide production, a nonribosomal peptide synthetase (NRPS) gene is commonly found as a main element of the cluster. Large multifunctional enzymes are encoded by members of this gene family that produce a broad spectrum of bioactive compounds. In this research, we applied genome-based identification of nonribosomal peptide biosynthetic gene clusters in the family *Ceratocystidaceae*. For this purpose, we used the whole genome sequences of species from the genera *Ceratocystis,* *Davidsoniella,* *Thielaviopsis*, *Endoconidiophora,* *Bretziella*, *Huntiella,* and *Ambrosiella*. To identify and characterize the clusters, different bioinformatics and phylogenetic approaches, as well as PCR-based methods were used. In all genomes studied, two highly conserved NRPS genes (one monomodular and one multimodular) were identified and their potential products were predicted to be siderophores. Expression analysis of two *Huntiella* species (*H. moniliformis* and *H. omanensis*) confirmed the accuracy of the annotations and proved that the genes in both clusters are expressed. Furthermore, a phylogenetic analysis showed that both NRPS genes of the *Ceratocystidaceae* formed distinct and well supported clades in their respective phylograms, where they grouped with other known NRPSs involved in siderophore production. Overall, these findings improve our understanding of the diversity and evolution of NRPS biosynthetic pathways in the family *Ceratocystidaceae*.

## 1. Introduction

Fungi produce an extensive variety of secondary metabolites (SMs) [1]. These small organic molecules differ from primary metabolites as they are not essential for growth in vitro. In fungi, one class of secondary metabolites, nonribosomal peptides (NRPs), are known to function in basic metabolism, as well as an array of other biological processes including cellular development and morphology, pathogenicity, and stress responses [1]. This diverse family of natural products has also received attention because of its medicinal uses as immunosuppressive drugs or antibiotics [2].

NRPs are small bioactive peptides that are not synthesized via the main ribosome-based translational process, but instead by serial reduction of proteinogenic and/or nonproteinogenic amino acids [3,4]. At least 500 different monomers containing hydroxy acids, fatty acids, and nonproteinogenic amino acids have been identified as NRP building blocks [5]. These building blocks play a role in both the structural adaptability and diversity of biological activities present among NRPs [5]. In filamentous fungi, the genes required for biosynthesis of these NRPs are often clustered together in the genome [6,7]. In such a cluster, one of the genes encode a large mono- or multimodular enzyme, referred to as a nonribosomal peptide synthetase (NRPS), which is responsible for producing the NRP backbone [3,4]. Other genes in the cluster encode proteins involved in modifying the NRP and regulating its production and function [7].

Despite the structural and chemical diversity of known NRPs, all NRPS modules contain at least three conserved domains [2,8]. These include an adenylation domain (A) to identify and activate the substrates, a thiolation domain (T) for covalent binding and amino acid transmission, and a condensation domain (C) for peptide bond formation. Hence, each NRPS module is responsible for the recognition (through the A domain) and integration of a single amino acid into the extending peptide chain. Therefore, NRPSs are often composed of multiple modules, depending on the complexity of the SM produced. An NRPS enzyme may also contain accessory domains such as a thioesterase (TE) needed for release of the newly synthesized peptide from the enzyme, as well as amino acid epimerisation (E) and N-methyl transferase (MT) domains for modifying the final product [5,9,10,11,12]. NRPS modules may further occur together with other SM biosynthesis gene clusters, such as those encoding polyketide synthases (PKS) to form hybrid PKS–NRPS enzymes [13].

Genome sequencing studies on filamentous fungi have exposed a surprisingly large number of SM biosynthesis genes and gene clusters, including many responsible for the production of NRPs [14,15,16,17,18]. For example, genome studies have been conducted for NRPS genes and clusters in fungi such as *Aspergillus fumigatus* [19], *Fusarium* species [20], and *Cochliobolus heterostrophus* [21]. Bioinformatics tools have also become more advanced and are able to efficiently and accurately identify SM biosynthesis gene clusters. Notable examples include SMURF (secondary metabolite unique regions finder) [22] and antiSMASH (antibiotics and secondary metabolite analysis shell) [23].

Previous studies of fungal SM biosynthesis clusters have revealed that the more conserved NRPSs, comprised mostly of mono and bimodular enzymes, normally function in basic cellular processes, including those essential for life [24]. By contrast, multimodular NRPSs are often involved in the production of SMs that are species-specific or strain-specific [25]. The latter are limited to fungi and are characterized by domain architectures which are less stable and synthesize NRPs with more niche-specific roles [25]. Phylogenetic analyses of the NRPS and PKS–NRPS hybrid enzymes have also revealed the involvement of duplication, divergence, and differential loss of genes, as well as horizontal gene transfer in their evolution [26,27]. However, Bushley and Turgeon [25] showed that several mono/bimodular subfamilies arose either prior to, or early in, the evolution of fungi. Despite the diversity of NRPS, NRPS-like, and PKS–NRPS hybrid enzymes encoded by fungi, fairly little is known about the SMs they produce [13].

The current study focuses on the NRPS gene clusters in the family *Ceratocystidaceae*. This taxon accommodates at least 11 genera (i.e., *Ambrosiella, Berkeleyomyces, Bretziella, Ceratocystis, Chalaropsis, Davidsoniella, Endoconidiophora, Huntiella, Phialophoropsis, Meredithiella*, and *Thielaviopsis*), many of which are plant pathogens and insect associates [28,29]. Various authors have suggested that SMs might play significant roles in the biology of these important fungi [30,31], but such compounds have been only identified in *Endoconidiophora resinifera* [32]. At the genomic level, the only SM biosynthesis gene clusters characterized in these fungi were those encoding polyketides [33]. The overall goal of this study was therefore to identify and characterize putative NRP biosynthesis clusters and genes in the *Ceratocystidaceae*. For this purpose, we used a comparative genomics approach to identify and compare NRPS genes and clusters among these genomes. We also used a phylogenetic approach to further identify the possible products of our clusters and to evaluate the evolution of these genes.

## 2. Materials and Methods

### 2.1. Genome Assemblies

The draft genome sequences of 23 isolates representing 22 *Ceratocystidaceae* species were used in this study (Table 1). These included seven *Ceratocystis* sensu stricto species (*C. fimbriata, C. harringtonii*, *C. manginecans*, *C. eucalypticola, C. albifundus, C. smalleyi,* and *C. platani*), five *Huntiella* species (*H. moniliformis*, *H. bhutanensis*, *H. omanensis*, *H. savannae,* and *H. decipiens*), three species of *Davidsoniella* (*D. virescens*, *D. australis,* and *D. neocalidoniae*), two *Thielaviopsis* species (*T. punctulata* and *T. musarum*) and two *Endoconidiophora* species (*E. laricicola* and *E. polonica*), as well as *C. adiposa*, *Berkeleyomyces basicola*, *Bretziella fagacearum,* and *Ambrosiella xylebori*. Apart from *D. australis* and *D. neocalidoniae*, all genome sequences were either published or available in the public domain (Table 1).

*Davidsoniella australis* isolate CMW2333 [28] and *D. neocalidoniae* isolate CMW26392 [34] were obtained from the culture collection (CMW) of the Forestry and Agricultural Biotechnology Institute (FABI), University of Pretoria, Pretoria, South Africa. Both isolates were grown on malt extract agar (MEA; 2% (*w/v*) Bacto™ malt extract (BD BioSciences, San Jose, CA, USA), and 2% (*w/v*) Difco™ agar (BD BioSciences, San Jose, CA, USA) at 25 °C for 14 days. High-quality DNA was extracted using a method employing CTAB (cetyl trimethylammonium bromide) [35], and sent for sequencing at the Central Analytical Facility (University of Stellenbosch, Stellenbosch, South Africa). For this purpose, the Ion-Torrent™ (Thermo Fisher Scientific, Johannesburg, South Africa) Ion S5™ system and Ion 530 Chip Kit were used to produce 400-nucleotide single reads. The raw sequence reads were filtered for quality and used for a de novo assembly in CLC Genomics Workbench v.11.0.1 (Qiagen Bioinformatics, Aarhus, Denmark) with default settings. All contigs longer than 500 bases were submitted to the nucleotide repository at the National Center for Biotechnology Information (NCBI; www.ncbi.nlm.nih.gov/genbank/). The completeness of all of the genomes included in this study was assessed using the Benchmarking Universal Single-Copy Orthologs (BUSCO) tool [36].

### 2.2. Identification of NRPS Genes and Clusters

To identify contigs that might contain NRPS clusters, each of the *Ceratocystidaceae* target genomes were submitted to the fungal version of antiSMASH v.4.0 [37] and the fungal-specific tool SMURF [22]. In cases where a cluster was predicted across multiple contigs, a reference assembly was used to confirm that these belonged to a single continuous sequence. To do this, the raw reads of the genome in question were mapped to a complete reference locus obtained from a close relative. For comparison, the repertoire of NRPS genes encoded by other *Sordariomycetes* were also determined using the antiSMASH and SMURF online tools. Full genome sequences for 16 representative *Sordariomycete* species were obtained from the NCBI database and the MycoCosm genomics resource [38] of the Joint Genome Institute’s fungal program [39] (Supplementary file S-1).

The *Ceratocystidaceae* contigs identified by antiSMASH and SMURF as putatively containing NRPS clusters were manually annotated. To do this, the identified and reconstructed contigs were annotated using the Web AUGUSTUS Service (http://bioinf.uni-greifswald.de/webaugustus/; [40]) based on *Fusarium graminearum* gene models with the default program parameters. The results were first analyzed for any nonribosomal peptide synthetase that could form the basis of a NRPS cluster by BLASTp searches against the GenBank database using the translated genes as query. Once this gene was identified, the predicted open reading frames (ORFs) present in 15 kilobases (Kb) of sequence upstream and downstream of the putative NRPS gene were searched for similarity to genes implicated in SM biosynthesis clusters using the results of the BLASTp search. The results of this analysis were used to infer the boundary of the NRPS cluster.

In order to further characterize the similarities between the *Ceratocystidaceae* NRPS sequences and those previously described, we examined the domain structure of the genes present in the defined cluster. Characterization of the domains present in these genes was done using the InterPro Scan tool (https://www.ebi.ac.uk/interpro/; [41]), the PKS/NRPS analysis website (http://nrps.igs.umaryland.edu/nrps) [42]), MOTIF search (http://www.genome.jp/tools/motif/) and NCBI’s Conserved Domain Database [43]. In addition, genes present in the defined cluster were examined for the presence of signature NRPS domains characteristic of the NRP biosynthesis enzymes [2,8] by making use of the online available PKS/NRPS Analysis website (http://nrps.igs.umaryland.edu). A putative NRPS gene was confirmed if it possessed at least one module containing all three of the conserved domains (i.e., A, T and C) [44]. The results for this analysis were also used to deduce the NRPS module organization.

### 2.3. Confirmation of Gene Order and Annotation

The gene order and content of the identified *Ceratocystidaceae* clusters was verified using a PCR-based approach. For this purpose, DNA was extracted from isolates that were grown on medium containing MEA, using the a DNeasy Plant Mini Kit (Qiagen, Carlsbad, CA, USA). A series of primers were designed to amplify the genes and intergenic sequences for five representatives of the family (*Ceratocystis manginecans*, CMW17570; *Thielaviopsis musarum*, CMW1546; *Endoconidiophora polonica,* CMW20930; *Huntiella bhutanensis,* CMW8217; *Davidsoniella virescens,* CMW17339; and *Bretziella fagacearum*, CMW2656). To design the primers, each identified gene was submitted to the online primer design software Primer3web [45] to design a forward and reverse primer. Details regarding the primers as well as PCR conditions used are presented in Supplementary file S-2. Amplicons were purified with Sephadex G50 columns (Sigma-Aldrich, Modderfontein, South Africa), and sequenced using the BigDye Terminator 3.1 cycle sequencing premix kit (Applied Biosystems, Foster City, CA, USA). The products were analyzed on an ABI PRISM 3300 Genetic Analyzer (Applied Biosystems, Foster City, CA, USA) at the Bioinformatics and Computational Biology Unit of the University of Pretoria. Consensus sequences were constructed with CLC Main Workbench v.9.1.1 (QIAGEN Bioinformatics, Aarhus, Denmark).

The annotations of the genes and clusters were evaluated using RNA-Seq data available from previous studies on *H. moniliformis* and *H. omanensis* [46]. The respective sets of sequence reads were quality filtered in CLC Genomics Workbench based on Phred quality scores, and reads with a score below 20 (Q ≤ 0.01) were discarded as described previously [46]. These data were then mapped to the original contigs containing NRPS gene clusters, by making use of the RNA-legacy tool in CLC Genomics Workbench using minimum similarity fractions of 0.8 and minimum length fractions of 0.5.

### 2.4. Phylogenetic Analysis of the Mono- and Multimodular NRPS Genes

The protein sequences of the A domain of the mono- and multimodular NRPS genes were used as a proxy to examine the evolutionary relationships of the putative NRPS orthologs. This is because the A domain represents the most conserved domain in fungal NRPS genes [47]. For this, datasets including sequences identified in this study, as well as those obtained from NCBI’s protein database, were subjected to phylogenetic analyses. The latter were identified by performing BLASTp searches against the GenBank database using the identified *Ceratocystidaceae* A domain NRPS proteins as query. Proteins identified with an expect value (E) ≤ 0.000001 were used in the analysis. Datasets were subjected to MAFFT (multiple alignment using fast Fourier transform) [48] using the E-INS-I function to allow for alignment with iterative refinement. Individual datasets were then used to construct neighbor-joining and maximum likelihood phylogenies based on suitable protein substitution models (LG + I + G + F for monomodular NRPS genes and JTT + I + G for multimodular NRPS genes) by making use of MEGA v.7 [49]. Branch support was estimated using bootstrap analysis of 1000 pseudo replicates and the same model parameters.

### 2.5. Fe–CAS Blue Agar Test

The universal siderophore assay using the iron–chrome azurol S (Fe–CAS) dye complex was performed as described by Milagres et al. [49,50]. This assay was used to test the ability of fungi in the *Ceratocystidaceae* to produce iron-binding complexes in the medium. For this purpose, the dye solution was made by dissolving 36.5 mg hexadecyltrimethyl-ammonium bromide (HDTMA) in 20 mL distilled water, and then gradually supplementing it with 25 mL of CAS solution (1.21 g/L) and 10 mL of an iron solution (containing 1 mM FeCl_3_·6H_2_0, 10 mM HCl). The dye solution was then added to 375 mL water containing piperazine-*N,N*′-bis(2-ethanesulfonic acid) (PIPES; 40 g/L; pH 6.8) and 20 g/L agar for the preparation of Fe–CAS blue agar medium in Petri plates. To prepare the Fe–CAS blue assay plates, half of the contents of a Petri dish containing MEA growth medium was removed and replaced with Fe–CAS blue agar medium. The tested fungal strains were inoculated onto the MEA half of the plate and incubated at 25 °C for three weeks in the dark until a colour change was observed in the Fe–CAS-containing half of the assay plates.

## 3. Results

### 3.1. Genome Assemblies

The quality-filtered Ion-Torrent sequence libraries for *D. australis* and *D. neocalidoniae* consisted of 4,353,100 and 3,307,125 reads, respectively. These were assembled into genomes that were respectively 38.6 Mb and 35.3 Mb in size. All contigs over 500 bp were deposited at DDBJ/ENA/GenBank under the accessions RHLR00000000 for *D. australis* and RHDR00000000 for *D. neocalidoniae*, with the respective versions described in this paper being RHLR01000000 and RHDR01000000. Based on the BUSCO analysis, these assemblies, as well as those obtained from NCBI and other sources, showed high levels of completeness (>96%), thus allowing for meaningful genomic comparisons (Table 1).

### 3.2. Identification of NRPS Genes and Clusters

**Figure 1:**
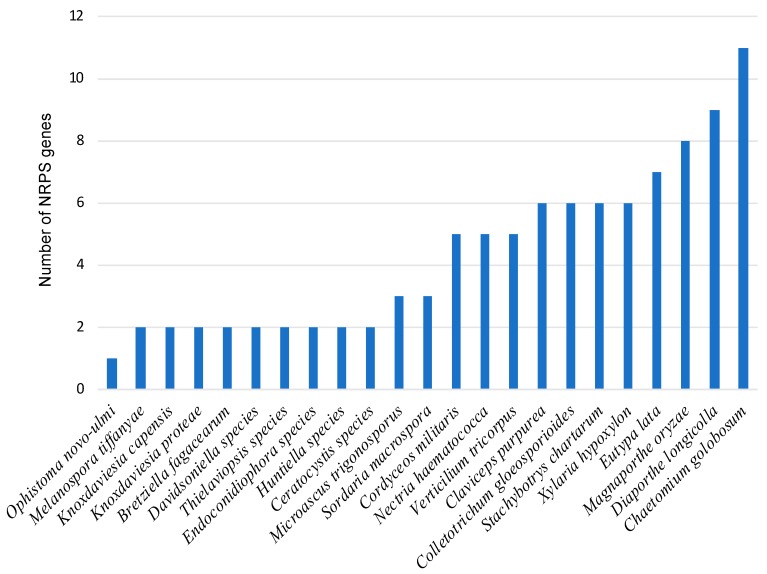
Number of nonribosomal peptide synthetase (NRPS) genes predicted from 40 *Sordariomycetes* genomes. The number of NRPS genes predicted by the online software SMURF (secondary metabolite unique regions finder) [22] and antibiotics and secondary metabolite analysis shell (antiSMASH) software [23] from 40 *Sordariomycetes* genomes from the National Center for Biotechnology Information (NCBI) and the Joint Genome Institute (JGI) are shown.

With the aid of SMURF and antiSMASH, contigs containing putative NRPS biosynthesis gene clusters were identified in both the *Ceratocystidaceae* and other *Sordariomycetes* genomes examined (Figure 1). Two putative NRPS biosynthesis gene clusters were identified in all the *Ceratocystidaceae* genomes examined, which included one multimodular and one monomodular NRPS gene cluster (Supplementary files S-3 and S-4). In two of the *Davidsoniella* species, namely *D. australis* and *D. neocaledoniae*, the genes of the monomodular clusters occurred on more than one contig. However, reference assemblies using the raw reads of these two fungi against the *D. virescens* assembly suggested that these genes are part of a single cluster in both species. The consensus sequences for the clusters in *D. neocaledoniae* and the *D. australis* have been deposited in NCBI with the accession numbers MK694917 and MK694918, respectively. When compared with other *Sordariomycetes*, the *Ceratocystidaceae* falls within the lower spectrum with regards to the number of NRPS biosynthesis gene clusters encoded. Among the fungi considered, *Chaetomium golobosum* (11 clusters) and *Diaporthe longicolla* (nine clusters) had the greatest number of such clusters.

**Figure 2:**
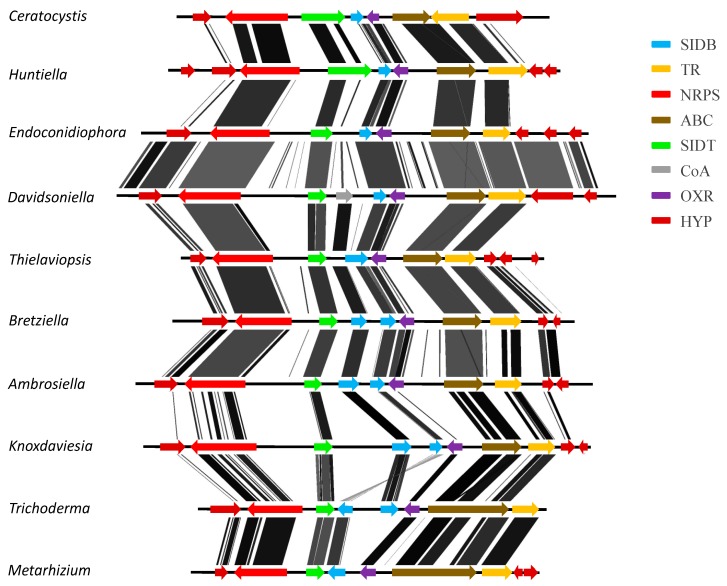
Comparison of the siderophore biosynthesis gene clusters of *Ceratocystidaceae*, *Trichoderma virens,* and *Metarhizium anisopliae*. OXR, oxidoreductase; TR, transporter; HYP, hypothetical protein; SIDB, siderophore biosynthesis protein; SIDT, siderophore transporter; ABC, ABC transporter; NRPS, nonribosomal peptide synthetase; CoA, co acetyl transferase. Shaded lines represent similarities between nucleotide sequences. The figure was generated using EASYFIG version 2.2.2 (http://mjsull.github.io/Easyfig/files.html) and the GenBank information for each cluster.

The gene content and order of the putative monomodular NRPS gene cluster was identical across all the *Ceratocystidaceae* genomes, and appeared to represent an extracellular-type siderophore biosynthesis cluster (Supplementary files S-3 and S-4). This is because the putative NRPS gene present in this cluster shared high similarity (≥90% sequence similarity) to genes present in extracellular-type siderophore biosynthesis clusters present in *Colletotrichum*, *Fusarium, Trichoderma*, and *Metarhizium* (Supplementary file S-5). In addition to the NRPS gene, this cluster also contained genes coding for a siderophore biosynthesis gene, a siderophore transporter, and an ABC-transporter (Figure 2).

**Figure 3:**
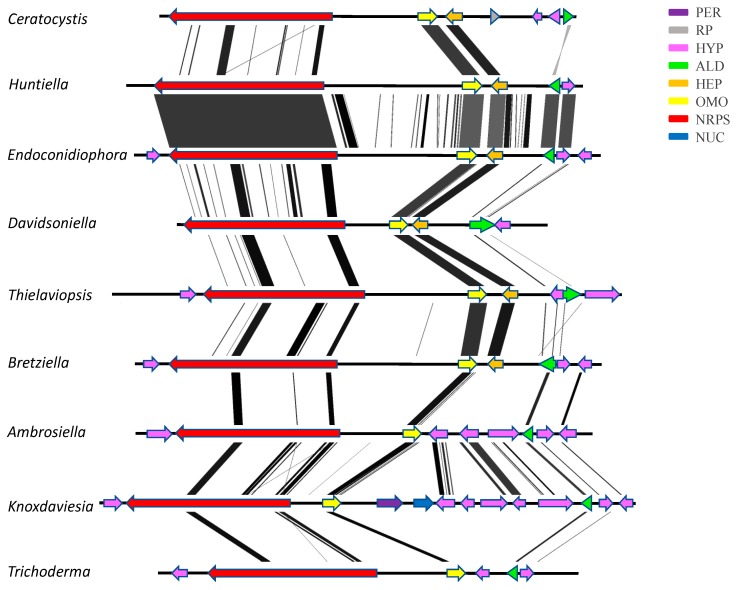
Comparison of the ferrichrome biosynthesis gene clusters of *Ceratocystidaceae* and *Trichoderma virens*. PER, peroxidase; RP, RNA polymerase transcription subunit; HYP, hypothetical protein; ALD, aldehyde dehydrogenase; HEP, endothiapepsin; OMO, orntithine monooxygenase; NRPS, nonribosomal peptide synthetase; NUC, nuclease. Shaded lines represent similarities between nucleotide sequences. The figure was generated using EASYFIG version 2.2.2 (http://mjsull.github.io/Easyfig/files.html) and the GenBank information for each cluster.

The putative multimodular NRPS gene cluster identified in the *Ceratocystidaceae* genomes (Supplementary files S-3 and S-4) also appeared to represente a siderophore biosynthesis cluster. This is because the NRPS gene located in this cluster shared a very high similarity (96–99%) to one encoding a ferricrocin synthetase (20). The cluster contained three complete NRPS modules, which resembled that of other known siderophore synthetases, specifically those of *Fusarium oxysporum*, *Colletotrichum gloesporioides*, *Scedosporium apiospermum,* and *Colletotrichum gloesporioides* (Supplementary file S-5). It was also flanked by a gene encoding an L-ornithine N5-monooxygenase with high similarity (88–99%) to that encoded near the ferricrocin biosynthesis cluster of *Colletotrichum gloesporioides*. The *Ceratocystidaceae* cluster was comprised of a NRPS, an L-ornithine N5-monooxygenase, endothiapepsin, two hypothetical protein coding genes, and a transcription factor (Figure 3).

**Figure 4:**
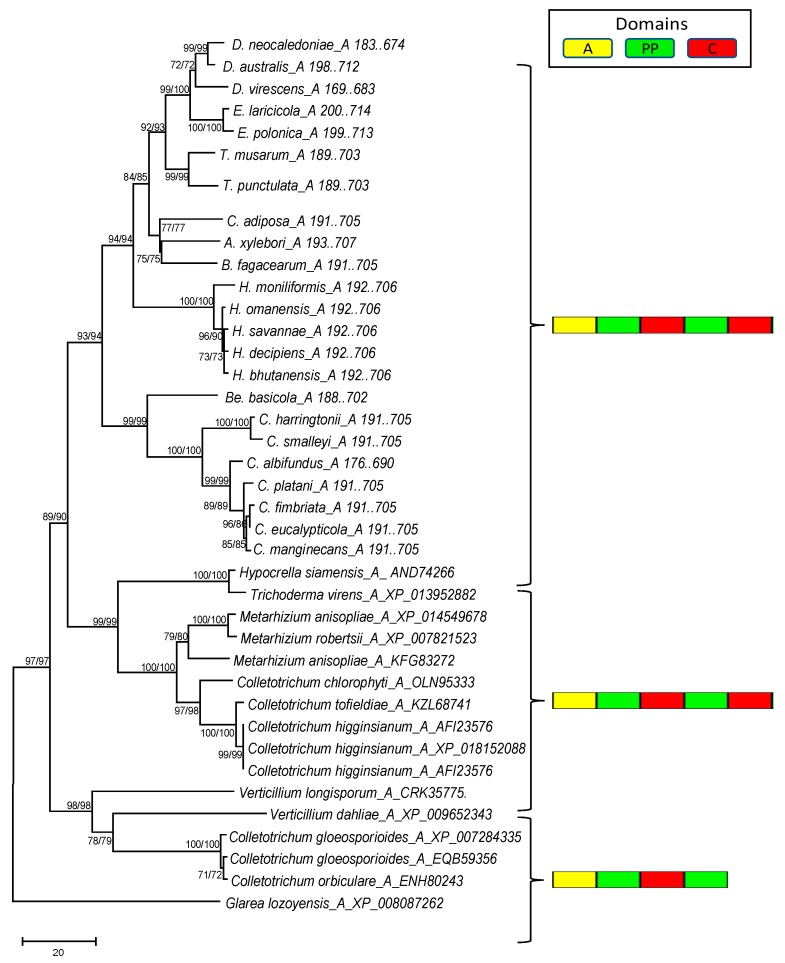
Phylogenetic relationships based on the inferred protein sequences of the A domain from the monomodular NRPS gene examined in this study. The panel to the right indicates the domain structure of the NRPS genes in the respective clusters. The tree was inferred using the neighbor-joining (NJ) method in MEGA v.7 with the LG + I + G + F model. Similar groupings were obtained using maximum likelihood (ML) analysis and the same model parameters. Branch support is indicated at the internodes (>50% bootstrap values based on a 1000 repeats) in the order NJ/ML.

**Figure 5:**
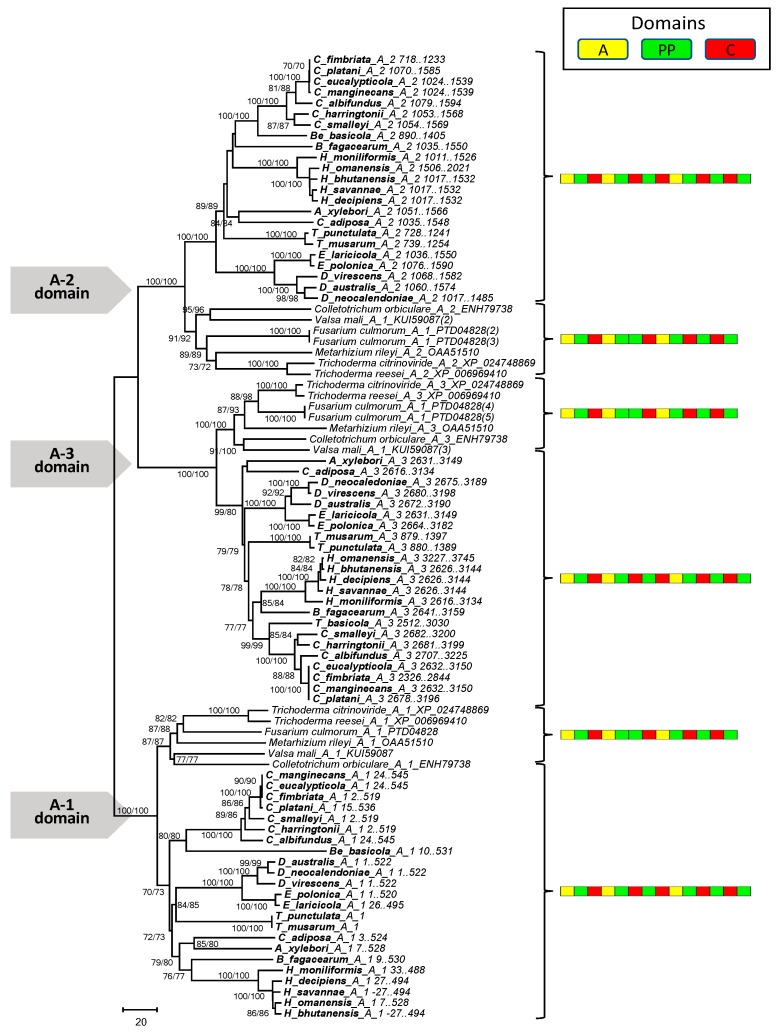
Phylogenetic relationships of taxa based on the inferred protein sequences of A domains of the multimodular NRPS genes examined. The panel to the right indicates the domain structure of the NRPS genes in the respective clusters. The tree was generated with the neighbor-joining (NJ) method in MEGA v.7 using the JTT + I + G model. Similar groupings were obtained using maximum likelihood (ML) analysis and the same model parameters. Branch support is indicated at the internodes (>50% bootstrap values based on a 1000 repeats) in the order NJ/ML.

Conserved domain analysis showed that the domain organization of the monomodular NRPS gene was highly conserved in all the *Ceratocystidaceae* genomes examined (see Figure 4 and Figure 5 below). These genes contained the adenylation, thiolation, and condensation domains in the order A–T–C–T–C, similar to the NRPS genes reported for *Trichoderma virens* (XP_013952882) and *Metarhizium anisopliae* (XP_014549678). In contrast to the monomodular NRPS genes, the domains in the multi-modular NRPS gene of the *Ceratocystidaceae* occurred in the order A–T–C–A–T–C–T–C–A–T–C–T–C–T (or slight variations of this). This was similar to those of *Colletotrichum orbiculare* (NCBI accession number ENH79738) and *Trichoderma reesei* (NCBI accession number XP_006969410). The domain structure in the *Ceratocystidaceae* multimodular NRPS genes was typical of the genes involved in the biosynthesis of ferricrocin-type siderophores (i.e., ferrichrome synthetase group) [51]. However, the *Ceratocystidaceae* multimodular NRPS gene differed in that they had a T–C next to the second A–T–C module, while the ferrichrome synthetase genes examined previously only had three complete A–T–C modules and a terminal T–C repeat [52].

### 3.3. Confirmation of Gene Order and Annotation

PCR amplification and sequencing of the genes and intergenic regions confirmed the gene content and order within each of the two clusters (Supplementary file S-2). In these analyses, lengths and sequences of the amplicons corresponded with those predicted from the respective assemblies. Analysis of the mapped RNA-seq data of *H. moniliformis* and *H. omanensis* confirmed the annotation of the clusters and genes while also confirming that these genes were expressed (Supplementary file S-6).

### 3.4. Phylogenetic Analysis of the Mono- and Multimodular NRPS Genes

The phylogenetic analysis revealed that the A domain of both the mono- and multimodular NRPS genes predicted in the genomes of *Ceratocystidaceae* formed distinct and well supported clades in their respective phylograms (Figure 4 and Figure 5). In the case of the monomodular NRPS (Figure 4), the *Ceratocystidaceae* clustered with the A domains of monomodular NRPS sequences from other Ascomycetes (e.g., *Trichoderma virens* (NCBI accession number XP_013952882) and *Metarhizium anisopliae* (NCBI accession number XP_014549678)) that are known to be involved in the production of siderophores [51].

For the multimodular NRPS domain A phylogeny (Figure 5), sequences separated into three groups, where each contained the sequences for one of the *Ceratocystidaceae* NRPS A domains, together with those of fungal NRPS genes that have been functionally shown to produce siderophores [51,53]. For example, the first A domain grouped with the first A domain in the NRPS genes of *Metarhizium rileyi* (NCBI accession number OAA51510), *Trichoderma citrinoviride* (NCBI accession number XP_024748869), *Trichoderma reesei* (NCBI accession number XP_006969410), *Fusarium culmorum* (NCBI accession number PTD04828), *Valsa mali* (NCBI accession number KUI59087), and *Colletotrichum orbiculare* (NCBI accession number ENH79738).

### 3.5. Fe–CAS Blue Agar Test

Based on our Fe–CAS blue assay, all of the *Ceratocystidaceae* isolates examined produced iron-binding compounds (Table 2). All the isolates grew normally on the MEA half of the medium, but produced characteristic pink to purple halos when they reached the Fe–CAS blue half of the medium. This indicated that the growth of the fungus was associated with removal of Fe from the Fe–CAS containing medium [49]. However, the individual species differed in the strength of their iron-binding ability as a range of colour changes (purple, pink, or purplish-red) were observed.

## 4. Discussion

This study is the first to explore the repertoire of NRPS biosynthetic gene clusters in the *Ceratocystidaceae*. The use of genome data, together with various bioinformatic tools, facilitated the identification of two such clusters in these fungi. In this regard, *Ceratocystidaceae* falls on the lower end of the spectrum of fungi encoding NRPS biosynthetic gene clusters [54]. The genomes of fungi such as *Colletotrichum gloeosporioides* and *Diaporthe longicolla* contain many more genes and clusters potentially responsible for the production of NRPs (e.g., they respectively encode 11 and nine putative NRPS genes).

The two NRPS biosynthetic gene clusters identified in the *Ceratocystidaceae* likely encode siderophores. This is mostly based on the similarity of the core gene sequences of the clusters to those of other known NRPSs, but gene knockout studies are needed for verification. Siderophores are low molecular weight, iron-binding molecules that enable iron uptake in microorganisms [55,56]. Most fungi utilize NRPS to produce hydroxamate-type siderophores that share the structural unit *N*^5^-acyl-*N*^5^-hydroxyornithine (i.e., fusarinines, coprogens, and ferrichromes) [57,58]. By contrast, certain Zygomycetes and bacteria utilize NRPS-independent mechanisms to produce polycarboxylate siderophores [59,60,61]. However, all known siderophores of the Ascomycetes are produced by NRPSs [60].

One of the predicted *Ceratocystidaceae* NRPS genes is monomodular and the other is multimodular. Different from other multimodular NRPSs that usually terminate with a condensation-like domain involved in releasing the final peptide, the *Ceratocystidaceae* multimodular NRPS gene terminates with a C–T domain. This is similar to what has been found for *Penicillium chrysogenum* (Gene ID, Pc13g05250) [16]. However, previous studies have also shown that the occurrence of C and T domains at the carboxy terminus may be characteristic of iterative NRPS [47]. Indeed, C and T domains were often found at the C terminus of the majority of *Aspergillus* and *Penicillium* NRPSs [47,62]. With regards to monomodular NRPS genes, the one encoded by the *Ceratocystidaceae* terminates with a C, while those of other fungi typically have a T terminal domain [25]. Interestingly, this domain architecture is also identified at the carboxy terminus of the *Ustilago maydis* NRPS gene, and the protein product functions to close the growing tripeptide ring of the siderophore [63].

The monomodular NRPS gene identified in the *Ceratocystidaceae* is likely involved in the production of an extracellular siderophore. This is because the putative NRPS gene existing on this cluster shared ≥90% sequence similarity to those present in the extracellular-type siderophore biosynthesis clusters of other Ascomycetes (e.g., *Fusarium, Metarhizium,* and *Trichoderma*) [53,64]. Along with the NRPS gene, this cluster is also comprised of other important genes related to extracellular siderophore production (e.g., genes encoding a siderophore transporter and an ABC-transporter). A homologous cluster has been reported in various fungi including *Trichoderma virens*, *Metarhizium anisopliae*, *Sordaria macrospora,* and *Fusarium graminearum* [53,64]. In *T. virens*, the same cluster is responsible for the biosynthesis of an extracellular siderophore involved during host infection [51], and gene deletion studies revealed its capacity to produce many additional secreted siderophores [53]. During plant infection, extracellular siderophores are thought to supply the fungus with iron as an essential nutrient in planta, thereby enhancing its virulence [65,66]. These types of siderophores are often implicated in pathogenicity or virulence of fungal pathogens [67].

The multimodular NRPS of the *Ceratocystidaceae* is likely responsible for the production of an intracellular siderophore. This gene is highly conserved among Ascomycetes and encodes a ferricrocin synthetase [68]. The first step in intracellular siderophore biosynthesis is the formation of *N*^5^-hydroxy-L-ornithine through *N*^5^-hydroxylation of L-ornithine [20]. This reaction is catalyzed by an L-ornithine-*N*^5^-monooxygenase. In most cases, the genes encoding this enzyme are located in the vicinity of an NRPS gene [20,69], and in the *Ceratocystidaceae* genomes examined, a homolog of it was located next to the NRPS gene. Similarly, in the genomes of *F. pseudograminearum* and *Verticillium dahliae,* homologs of these two genes are positioned next to each other [20]. The role of this cluster in ferricrocin biosynthesis has been confirmed experimentally [54]. Knock out studies in *A. fumigatus* of the gene encoding L-ornithine *N*^5^-monooxygenase revealed that the transformants were unable to synthesize ferricrocin and fusarinine C siderophores [66,70]. Ferricrocin siderophores appear to be essential elements for the growth and survival of many fungi. In *Cochliobolus heterostrophus* and *Fusarium graminearum,* ferricrocin also has been shown as essential for sexual development [71]. Additionally, in *Trichoderma virens* ferricrocin is needed for conidial development [72] and in *A. fumigatus* it is an intracellular iron transporter involved in the distribution of iron during cellular development [73].

Similar to other NRPS enzymes, the *Ceratocystidaceae* NRPS contains three specifically recognized domains (A, T, and C) for peptide bond formation [2,8]. In some cases, the T–C units lacking an A domain may be functional by stimulating nonadjacent A domains [74]. T–C units, lacking an associated A domain, may be created either through independent duplication of T–C units or through loss of an associated A domain from a complete A–T–C module. Our results are congruent with the hypothesis of Schwecke and colleagues [74] of a hexamodular origin with six complete A–T–C units, following loss of separate A domains, for the ferrichrome synthetase [52]. For example, the *Ceratocystidaceae* multimodular NRPSs have six T domains, while they have only three A domains. To the best of our knowledge, the mechanisms regulating iterative usage of NRPS domains are unknown. It is also likely that proteins with similar domain structures most likely produce very similar secondary metabolites.

Given that *Ceratocystidaceae* likely encode two NRPS-dependent siderophores, our results correspond to those shown for *F. graminearum*, which also encoded one intracellular and one extracellular siderophore [65,71]. Various aspects of our results also point towards biological functionality of the two NRPS biosynthetic gene clusters found in *Ceratocystidaceae*. These NRPS genes are highly conserved and have high similarity to those of fungi known to produce siderophores, which would be expected for loci involved in essential biological functions [52,74]. The same is also true for their overall cluster structures and gene content. Additionally, we have found evidence in previously published RNA-seq data [46] that almost all of the genes in both of the clusters are expressed in at least two members of the family. Finally, isolates of *Ceratocystidaceae* exhibited siderophore-like activity by binding Fe when they were grown on medium containing the Fe–CAS dye complex [50]. Our future research would therefore seek to reveal the roles of NRPS genes and clusters in the overall biology, ecology, and host-associations of the *Ceratocystidaceae*.

## Figures and Tables

**Table 1:** Isolate numbers, GenBank accession numbers, and completeness information for the *Ceratocystidaceae* assemblies included in this study.

Species ^a^	Isolate Number ^b^	GenBank Accession Number	BUSCO Completeness Scores ^c^			
			Complete and Single Copy (%)	Complete and Duplicated (%)	Fragmented (%)	Missing (%)
*B. fagacearum*	CMW 2656	MKGJ00000000	95.2	0	3.1	1.7
*C. adiposa*	CMW 2573	LXGU00000000	99.3	0	0.3	0.4
*C. albifundus*	CMW 13980	JSSU000000000	97.6	0	1.4	1
*C. eucalypticola*	CMW 11536	LJOA00000000	97.9	0	1.4	0.7
*C. fimbriata*	CMW 15049	APWK00000000	91.0	0.3	1.4	7.6
*C. harringtonii*	CMW 14789	MKGM0000000	97.9	0	0.7	1.4
*C. manginecans*	CMW 17570	JJRZ000000000	97.2	0.3	1.7	1.1
*C. platani*	CF0	LBBL00000000	99.3	0	0.4	0
*C. smalleyii*	CMW 14800	NETT01000000	99	0	0.7	0.3
*D. virescens*	CMW 17339	LJZU000000000	97.6	0	1.4	1
*E. laricicola*	CMW 20928	LXGT00000000	98.9	0.3	0.7	0.4
*E. polonica*	CMW 20930	LXKZ00000000	97.6	0	1.4	1
*H. bhutanensis*	CMW 8217	MJMS00000000	96.2	0.3	2.1	1.7
*H. decipiens*	CMW 30855	NETU00000000	96.9	0.3	2.1	1
*H. moniliformis*	CMW 10134	JMSH00000000	94.8	0.3	3.4	1.8
*H. omanensis*	CMW 11056	JSUI000000000	88.6	0.3	7.9	3.5
*H. savannae*	CMW 17300	LCZG00000000	96.2	1.7	2.1	1.7
*T. musarum*	CMW 1546	LKBB00000000	98.6	0	1.4	0
*T. punctulata* 1	BPI 893173	LAEV00000000	99.3	0.7	0.7	0
*T. punctulata* 2	CMW 1032	MJMR01000000	99.7	0.7	0.3	0
*D. neocaledoniae*	CMW 225392	RHDR00000000	96.9	0	2	7
*D. australis*	CMW 2333	RHLR00000000	95.5	0	5	8
*A. xylebori*	CBS 110.61	PCDO00000000	98.6	0	4	0
*K. capensis*	CBS 139037	LNGK00000000	99.3	0	2	0
*K. proteae*	CBS 140089	LNGL00000000	97.6	0	1	6
*Be. basicola*	CMW 49352	PJAC00000000	99.0	0	0	3

^a^ The two isolates of *T. punctulata* included here are indicated with the digits “1” and “2”. ^b^ Isolates with CMW numbers may be obtained from the culture collection of the Tree Protection Cooperative Programme (TPCP), Forestry and Agricultural Biotechnology Institute (FABI), University of Pretoria, Pretoria, South Africa. Those with CFO and BPI numbers may be obtained from respectively Istituto per la Protezione delle Piante (Consiglio Nazionale delel Ricerche Florence, Italy) and the US National Fungus Collections Systematic Botany and Mycology Laboratory (Maryland, U.S.A.). ^c^ The completeness of each genome assembly and annotation was evaluated with the Benchmarking Universal Single-Copy Orthologs (BUSCO) v.2 tool by employing the set of single copy genes conserved among all fungi (36). The gene matches that were only partially recovered are classified as “fragmented”, and BUSCO groups for which there were no matches that pass the tests of orthology are classified as “missing”.

**Table 2:** Evaluation of siderophore production by different *Ceratocystidaceae* representatives included in this study using the Fe–CAS (iron–chrome azurol S) blue agar universal test (49).

Species	Growth (days) ^a^	CAS Reaction	CAS-Blue Agar (Colour Change)
*B. fagacearum*	14	+++	Purple
*C. fimbriata*	13	+	Pink
*H. moniliformis*	7	++	Purple
*T. punctulata*	5	++	Purplish red
*D. virescens*	13	+	Pink
*E. polonica*	10	+	Pink
*C. adiposa*	5	++	Purplish red

^a^ Time (days) required for the fungal mycelium to cover the non Fe–CAS half of the plate.

## Supplementary Materials

The supplementary material is available: https://www.mdpi.com/2073-4425/10/5/328/s1. Supplementary file S1. Genome sequence information for the 16 *Sordariomycetes* representative included in this study. Secondary metabolite unique regions finder (SMURF; www.jcvi.org/smurf/; (22) was used to predict NRPS genes from the *Sordariomycetes* genomes available from the Joint Genome Institute (JGI; https://jgi.doe.gov/our-science/science-programs/fungal-genomics/) and the National Centre for Biotechnology Information (NCBI; http://blast.ncbi.nlm.nih.gov/). Supplementary file S2. To confirm the order of genes within the different NRPS clusters identified, a PCR-based approach was used. For each cluster type, primers were designed that allow amplification of individual genes, as well as the regions between them. Correlation between predicted and observed fragment sizes were used as evidence that the specific cluster was correctly assembled. Supplementary file S3. Blast hits for putative *Ceratocystidaceae* NRPS biosynthetic cluster genes. For confirmation of the antiSMASH results, we utilized secondary metabolite unique regions finder (SMURF; www.jcvi.org/smurf/; (22). For this purpose, genes that were 15 Kb upstream and downstream of the identified NRPS genes were retrieved and submitted to the BLASTp server at the National Center for Biotechnology Information (NCBI, ftp://ftp.ncbi.nih.gov/blast/) for identification. Supplementary file S4. The tables below show the predicted nonribosomal peptide synthetase (NRPS) gene clusters predicted by SMURF (secondary metabolite unique regions finder) (22; 37). Supplementary file S5. Top 10 BLASTp hits for each NRPS sequence. BLASTp search was done against the non-redundant protein sequences in the National Centre for Biotechnology Information database (NCBI; http://blast.ncbi.nlm.nih.gov/), and the top 10 hits are indicated with species where they are found, E-value, percent sequence identity, and coverage. Supplementary file S6. Mapping of RNA reads to different genes of the NRPS gene clusters of *C. fimbriata*, *H. moniliformis,* and *H. omanensis*. This included the genes of the monomodular NRPS gene clusters: A, hypothetical protein; B, nonribosomal peptide synthetase; C, siderophore transporter; D, siderophore biosynthesis; E, oxidoreductase; F, ABC transporter; and G, transporter. It also included mapping of RNA reads to different genes of multimodular NRPS gene clusters of *C. fimbriata*, *H. moniliformis,* and *H. omanensis*. A, nonribosomal peptide synthetase; B, orntithine monooxygenase; C, endothiapepsin; D, RNA polymerase transcription subunit; and E, aldehyde dehydrogenase. Reads in green and red represent forward and reverse orientation, respectively.

## References

[1] Keller N.P., Turner G., Bennett J.W. (2005). Fungal secondary metabolism—from biochemistry to genomics. Nat. Rev. Microbiol..

[2] Hoffmeister D., Keller N.P. (2007). Natural products of filamentous fungi, enzymes, genes, and their regulation. Nat. Prod. Rep..

[3] Walsh C.T. (2007). The chemical versatility of natural-product assembly lines. Acc. Chem. Res..

[4] Finking R., Marahiel M.A. (2004). Biosynthesis of nonribosomal peptides. Annu. Rev. Microbiol..

[5] Caboche S., Pupin M., Leclère V., Fontaine A., Jacques P., Kucherov G. (2007). NORINE, a database of nonribosomal peptides. Nucleic Acids Res..

[6] Keller N.P., Hohn T.M. (1997). Metabolic pathway gene clusters in filamentous fungi. Fungal Genet. Biol..

[7] Soukup A.A., Keller N.P., Wiemann P. (2016). Enhancing nonribosomal peptide biosynthesis in filamentous fungi. Nonribosomal Peptide and Polyketide Biosynthesis.

[8] Von Döhren H. (2004). Biochemistry and general genetics of nonribosomal peptide synthetases in fungi. Molecular Biotechnolgy of Fungal Beta-Lactam Antibiotics and Related Peptide Synthetases.

[9] Grünewald J., Marahiel M.A. (2006). Chemoenzymatic and template-directed synthesis of bioactive macrocyclic peptides. Microbiol. Mol. Biol. Rev..

[10] Mootz H.D., Schwarzer D., Marahiel M.A. (2002). Ways of assembling complex natural products on modular nonribosomal peptide synthetases. ChemBioChem.

[11] Hur G.H., Vickery C.R., Burkart M.D. (2012). Explorations of catalytic domains in non-ribosomal peptide synthetase enzymology. Nat. Prod. Rep..

[12] Keating T.A., Ehmann D.E., Kohli R.M., Marshall C.G., Trauger J.W., Walsh C.T. (2001). Chain termination steps in nonribosomal peptide synthetase assembly lines, directed acyl-S-enzyme breakdown in antibiotic and siderophore biosynthesis. ChemBioChem.

[13] Fisch K. (2013). Biosynthesis of natural products by microbial iterative hybrid PKS–NRPS. RSC Adv..

[14] Galagan J.E., Calvo S.E., Cuomo C., Ma L.-J., Wortman J.R., Batzoglou S., Lee S.-I., Baştürkmen M., Spevak C.C., Clutterbuck J. (2005). Sequencing of *Aspergillus nidulans* and comparative analysis with *A. fumigatus* and *A. oryzae*. Nature.

[15] Machida M., Asai K., Sano M., Tanaka T., Kumagai T., Terai G., Kusumoto K.-I., Arima T., Akita O., Kashiwagi Y. (2005). Genome sequencing and analysis of *Aspergillus oryzae*. Nature.

[16] Van Den Berg M.A., Albang R., Albermann K., Badger J.H., Daran J.-M., Driessen A.J., Garcia-Estrada C., Fedorova N.D., Harris D.M., Heijne W.H. (2008). Genome sequencing and analysis of the filamentous fungus *Penicillium chrysogenum*. Nat. Biotechnol..

[17] Pel H.J., de Winde J.H., Archer D.B., Dyer P.S., Hofmann G., Schaap P.J., Turner G., de Vries R.P., Albang R., Albermann K. (2007). Genome sequencing and analysis of the versatile cell factory *Aspergillus niger* CBS 513.88. Nat. Biotechnol..

[18] Nierman W.C., Pain A., Anderson M.J., Wortman J.R., Kim H.S., Arroyo J., Berriman M., Abe K., Archer D.B., Bermejo C. (2005). Genomic sequence of the pathogenic and allergenic filamentous fungus *Aspergillus fumigatus*. Nature.

[19] Stack D., Neville C., Doyle S. (2007). Nonribosomal peptide synthesis in *Aspergillus fumigatus* and other fungi. Microbiology.

[20] Tobiasen C., Aahman J., Ravnholt K., Bjerrum M., Grell M., Giese H. (2007). Nonribosomal peptide synthetase (NPS) genes in *Fusarium graminearum*, *F. culmorum* and *F. pseudograminearium* and identification of NPS2 as the producer of ferricrocin. Curr. Genet..

[21] Lee B.-N., Kroken S., Chou D.Y., Robbertse B., Yoder O., Turgeon B.G. (2005). Functional analysis of all nonribosomal peptide synthetases in *Cochliobolus heterostrophus* reveals a factor, NPS6, involved in virulence and resistance to oxidative stress. Eukaryot. Cell.

[22] Khaldi N., Seifuddin F.T., Turner G., Haft D., Nierman W.C., Wolfe K.H., Fedorova N.D. (2010). SMURF, genomic mapping of fungal secondary metabolite clusters. Fungal Genet. Biol..

[23] Medema M.H., Blin K., Cimermancic P., de Jager V., Zakrzewski P., Fischbach M.A., Weber T., Takano E., Breitling R. (2011). antiSMASH, rapid identification, annotation and analysis of secondary metabolite biosynthesis gene clusters in bacterial and fungal genome sequences. Nucleic Acids Res..

[24] Pöggeler S., Wöstemeyer J. (2011). Evolution of Fungi and Fungal-Like Organisms.

[25] Bushley K.E., Turgeon B.G. (2010). Phylogenomics reveals subfamilies of fungal nonribosomal peptide synthetases and their evolutionary relationships. BMC Evol. Biol..

[26] Kroken S., Glass N.L., Taylor J.W., Yoder O., Turgeon B.G. (2003). Phylogenomic analysis of type I polyketide synthase genes in pathogenic and saprobic ascomycetes. Proc. Natl. Acad. Sci. USA.

[27] Schmitt I., Lumbsch H.T. (2009). Ancient horizontal gene transfer from bacteria enhances biosynthetic capabilities of fungi. PLoS ONE.

[28] De Beer Z.W., Duong T., Barnes I., Wingfield B.D., Wingfield M.J. (2014). Redefining *Ceratocystis* and allied genera. Stud. Mycol..

[29] Nel W., Duong T., Wingfield B.D., Wingfield M.J., De Beer Z. (2018). A new genus and species for the globally important, multihost root pathogen *Thielaviopsis basicola*. Plant Pathol..

[30] Gremaud G., Tabacchi R. (1996). Relationship between the fungus *Ceratocystis fimbriata* coffea and the canker disease of the coffee tree. Phytochemistry.

[31] Koch W.-G., Sinnwell V. (1987). Isopulegol from liquid cultures of the fungus Ceratocystis coerulescens (Ascomycotina). Zeitschrift für Naturforschung C.

[32] Loppnau P., Tanguay P., Breuil C. (2004). Isolation and disruption of the melanin pathway polyketide synthase gene of the softwood deep stain fungus *Ceratocystis resinifera*. Fungal Genet. Biol..

[33] Sayari M., Steenkamp E.T., van der Nest M.A., Wingfield B.D. (2018). Diversity and evolution of polyketide biosynthesis gene clusters in the *Ceratocystidaceae*. Fungal Biol..

[34] Van Wyk M., Wingfield B.D., Clegg P., Wingfield M.J. (2009). *Ceratocystis larium* sp. nov.; a new species from *Styrax benzoin* wounds associated with incense harvesting in Indonesia. Persoonia.

[35] Plaza G., Upchurch R., Brigmon R., Whitman W., Ulfig K. (2004). Rapid DNA extraction for screening soil filamentous fungi using pcr amplification. Pol. J. Environ. Stud..

[36] Simão F.A., Waterhouse R.M., Ioannidis P., Kriventseva E.V., Zdobnov E.M. (2015). BUSCO, assessing genome assembly and annotation completeness with single-copy orthologs. Bioinformatics.

[37] Blin K., Wolf T., Chevrette M., Lu X., Schwalen C., Kautsar S., Suarez Duran H., De Los Santos E., HU K., Nave M. (2017). antiSMASH 4.0—Improvements in chemistry prediction and gene cluster boundary identification. Nucleic Acids Res..

[38] Grigoriev I., Nikitin R., Haridas S., Kuo A., Ohm R., Otillar R., Riley R., Salamov A., Zhao X., Korzeniewski F. (2014). MycoCosm portal, gearing up for 1000 fungal genomes. Nucleic Acids Res..

[39] Grigoriev I., Cullen D., Goodwin S., Hibbett D., Jeffries T., Kubicek C., Kuske C., Magnuson J., Martin F., Spatafora J. (2011). Fueling the future with fungal genomics. Mycology.

[40] Stanke M., Tzvetkova A., Morgenstern B. (2006). AUGUSTUS at EGASP, using EST, protein and genomic alignments for improved gene prediction in the human genome. Genome Biol..

[41] Zdobnov E., Apweiler R. (2001). InterProScan—An integration platform for the signature-recognition methods in InterPro. Bioinformatics.

[42] Bachmann B., Ravel J. (2009). In aIlico prediction of microbial secondary metabolic pathways from DNA sequence data. Methods Enzymol..

[43] Marchler-Bauer A., Lu S., Anderson J.B., Chitsaz F., Derbyshire M.K., De Weese-Scott C., Fong J.H., Geer L.Y., Geer R.C., Gonzales N.R. (2010). CDD, a Conserved Domain Database for the functional annotation of proteins. Nucleic Acids Res..

[44] Rausch C., Hoof I., Weber T., Wohlleben W., Huson D.H. (2007). Phylogenetic analysis of condensation domains in NRPS sheds light on their functional evolution. BMC Evol. Biol..

[45] Untergasser A., Cutcutache I., Koressaar T., Ye J., Faircloth B., Remm M., Rozen S. (2012). Primer3—new capabilities and interfaces. Nucleic Acids Res..

[46] Wilson A.M., van der Nest M.A., Wilken P.M., Wingfield M.J., Wingfield B.D. (2018). Pheromone expression reveals putative mechanism of unisexuality in a saprobic ascomycete fungus. PLoS ONE.

[47] Cramer R.A., Stajich J.E., Yamanaka Y., Dietrich F.S., Steinbach W.J., Perfect J.R. (2006). Phylogenomic analysis of non-ribosomal peptide synthetases in the genus *Aspergillus*. Gene.

[48] Katoh K., Misawa K., Kuma K., Miyata T. (2002). MAFFT, a novel method for rapid multiple sequence alignment based on fast Fourier transform. Nucleic Acids Res..

[49] Kumar S., Stecher G., Tamura K. (2016). MEGA7, molecular evolutionary genetics analysis version 7.0 for bigger datasets. Mol. Biol. Evol..

[50] Milagres A.M., Machuca A., Napoleao D. (1999). Detection of siderophore production from several fungi and bacteria by a modification of chrome azurol S (CAS) agar plate assay. J. Microbiol. Methods.

[51] Zeilinger S., Gruber S., Bansal R., Mukherjee P.K. (2016). Secondary metabolism in *Trichoderma*—Chemistry meets genomics. Fungal Biol. Rev..

[52] Bushley K.E., Ripoll D.R., Turgeon B.G. (2008). Module evolution and substrate specificity of fungal nonribosomal peptide synthetases involved in siderophore biosynthesis. BMC Evol. Biol..

[53] Mukherjee P.K., Horwitz B.A., Herrera-Estrella A., Schmoll M., Kenerley C.M. (2013). *Trichoderma* research in the genome era. Annu. Rev. Phytopathol..

[54] Mukherjee P.K., Horwitz B.A., Kenerley C.M. (2012). Secondary metabolism in *Trichoderma*—A genomic perspective. Microbiology.

[55] Winkelmann G., Hawksworth D.L. (1991). Importance of siderophores in fungal growth, sporulation and spore germination. Frontiers in Mycology.

[56] Neubauer U., Nowack B., Furrer G., Schulin R. (2000). Heavy metal sorption on clay minerals affected by the siderophore desferrioxamine B. Environ. Sci. Technol..

[57] Leong J. (1986). Siderophores, their biochemistry and possible role in the biocontrol of plant pathogens. Annu. Rev. Phytopathol..

[58] Renshaw J.C., Robson G.D., Trinci A.P., Wiebe M.G., Livens F.R., Collison D., Taylor R.J. (2002). Fungal siderophores, structures, functions and applications. Mycol. Res..

[59] Thieken A., Winkelmann G. (1992). Rhizoferrin, a complexone type siderophore of the mocorales and entomophthorales (Zygomycetes). FEMS Microbiol. Lett..

[60] Sørensen J., Knudsen M., Hansen F., Olesen C., Fuertes P., Lee T., Sondergaard T., Pedersen C., Brodersen D., Giese H. (2014). Fungal NRPS-Dependent Siderophores, from Function to Prediction.

[61] Kadi N., Challis G. (2009). Siderophore biosynthesis, a substrate specificity assay for nonribosomal peptide synthetase-independent siderophore synthetases involving trapping of acyl-adenylate intermediates with hydroxylamine. Methods Enzymol..

[62] Gibson D., Donzelli B., Krasnoff S., Keyhani N. (2014). Discovering the secondary metabolite potential encoded within entomopathogenic fungi. Nat. Prod. Rep..

[63] Yuan W.M., Gentil G.D., Budde A.D., Leong S.A. (2001). Characterization of the *Ustilago maydis* sid2 gene, encoding a multidomain peptide synthetase in the ferrichrome biosynthetic gene cluster. J. Bacteriol..

[64] Hansen B.G., Mnich E., Nielsen K.F., Nielsen J.B., Nielsen M.T., Mortensen U.H., Larsen T.O., Patil K.R. (2012). A natural fusion of a cytochrome P450 and a hydrolase is involved in mycophenolic acid biosynthesis. Appl. Environ. Microbiol..

[65] Oide S., Moeder W., Krasnoff S., Gibson D., Haas H., Yoshioka K., Turgeon B.G. (2006). NPS6, encoding a nonribosomal peptide synthetase involved in siderophore-mediated iron metabolism, is a conserved virulence determinant of plant pathogenic ascomycetes. Plant Cell.

[66] Hissen A.H., Wan A.N., Warwas M.L., Pinto L.J., Moore M.M. (2005). The *Aspergillus fumigatus* siderophore biosynthetic gene sidA, encoding L-ornithine N5-oxygenase, is required for virulence. Infect. Immun..

[67] Gerwien F., Skrahina V., Kasper L., Hube B., Brunke S. (2017). Metals in fungal virulence. FEMS Microbiol. Rev..

[68] Hof C., Eisfeld K., Welzel K., Antelo L., Foster A., Anke H. (2007). Ferricrocin synthesis in *Magnaporthe grisea* and its role in pathogenicity in rice. Mol. Plant Pathol..

[69] Mei B., Budde A.D., Leong S.A. (1993). sid1, a gene initiating siderophore biosynthesis in *Ustilago maydis*, molecular characterization, regulation by iron, and role in phytopathogenicity. Proc. Natl. Acad. Sci. USA.

[70] Schrettl M., Bignell E., Kragl C., Joechl C., Rogers T., Arst H.N., Haynes K., Haas H. (2004). Siderophore biosynthesis but not reductive iron assimilation is essential for *Aspergillus fumigatus* virulence. J. Exp. Med..

[71] Oide S., Krasnoff S.B., Gibson D.M., Turgeon B.G. (2007). Intracellular siderophores are essential for ascomycete sexual development in heterothallic *Cochliobolus heterostrophus* and homothallic *Gibberella zeae*. Eukaryot. Cell.

[72] Mukherjee P., Hurley J., Taylor J., Puckhaber L., Lehner S., Druzhinina I., Schumacher R., Kenerley C. (2018). Ferricrocin, the intracellular siderophore of *Trichoderma virens*, is involved in growth, conidiation, gliotoxin biosynthesis and induction of systemic resistance in maize. Biochem. Biophys. Res. Commun..

[73] Wallner A., Blatzer M., Schrettl M., Sarg B., Lindner H., Haas H. (2009). Ferricrocin, a siderophore involved in intra-and transcellular iron distribution in *Aspergillus fumigatus*. Appl. Environ. Microbiol..

[74] Schwecke T., Göttling K., Durek P., Dueñas I., Käufer N.F., Zock-Emmenthal S., Staub E., Neuhof T., Dieckmann R., von Döhren H. (2006). Nonribosomal peptide synthesis in *Schizosaccharomyces pombe* and the architectures of ferrichrome-type siderophore synthetases in fungi. Chembiochem.

