# Supplementary material for: Distribution and Evolution of Nonribosomal Peptide Synthetase Gene Clusters in the Ceratocystidaceae
Source: Genes (Basel). 2019 Apr 30;10(5):328. doi: 10.3390/genes10050328 (PMC6563098; doi:10.3390/genes10050328)
Supplement: Supplementary file 1 [file genes-10-00328-s001.zip › Supplementary Files/Supplementary file S2 Feb 2019.docx]

**SUPPLEMENTARY FILE S2**

*(Sayari et al - Ceratocystidaceae Nonribosomal peptide synthetase gene clusters)*

To confirm the order of genes within the different NRPS clusters identified, a PCR-based approach was used. For each cluster type, primers were designed that allow amplification of individual genes, as well as the regions between them. Correlation between predicted and observed fragment sizes were used as evidence that the specific cluster was correctly assembled.

For these PCRs, DNA was extracted from the five representative isolates: *Ceratocystis manginecans* (CMW17570), *Thielaviopsis musarum* (CMW1546), *Endoconidiophora polonica* (CMW20930), *Huntiella bhutanensis* (CMW8217) *Davidsoniella virescens* (CMW17339) and *Bretziella fagacearum* (CMW2656). This was done using 14-day-old fungal cultures grown at room temperature on malt-extract-Agar (MEA; Merck) medium and the DNeasy Plant Mini Kit (Qiagen, Carlsbad, CA, USA).

The table below lists the primer sequences and expected amplicon sizes for all of the PCRs conducted in this study. Each PCR mixture contained 2.5Mmol MgCl_2_, 150μM of each dNTP, 0.1μmol of each primer, 1U of *Taq* polymerase and 10X reaction buffer (Roche Applied Science, Mannheim, Germany) in a total volume of 25μl. Amplification was carried out with the GeneAmp PCR system 9700 (Applied biosystems) with initial denaturation 94°C for 4min, followed by 35 cycles of 94°C for 30 sec, 60°C for 30 sec, 72°C for 1 min, and a final extension step at 72°C for 15 min. The sizes of the resulting PCR products were then estimated by making use of 1% (w/v) agarose (whiteheads scientific, South Africa) gel electrophoresis, GelRed™ (Biotium, Inc., Fremont, California) nucleic acid staining and an UV-transilluminator.

Table 1. Sequences, annealing temperatures and GC content for the primers used in this study.

A. Primers for individual genes in the *Ceratocystis* monomodular group

| Primer | Primer sequence (5'→3') | Annealing temperature (⸰C) | GC content (%) |
| --- | --- | --- | --- |
| Hypothetical (F) | CAACAACAGAAGGAATTTCG | 55 | 40 |
| Hypothetical (R) | GGCCTAGTACTTTTTGAATG | 54 | 40 |
| Hypothetical-NRPS (F) | GAAACCCTCTTTTTTGCTCT | 56 | 40 |
| Hypothetical-NRPS (R) | GGATGGAGTTTGAGGTGTTG | 57 | 50 |
| NRPS (F) | GCTTTCAACACCTCAAACTC | 56 | 45 |
| NRPS (R) | TAAACTACGATGCCTCTGTC | 55 | 45 |
| NRPS-Acyl CoA (F) | GAGGCATCGTAGTTTATGTA | 54 | 40 |
| NRPS-Acyl CoA (R) | CCGTACAAAACTGATAGCTT | 55 | 40 |
| Acyl CoA (F) | ACTCAGACACGCATTGATAC | 57 | 45 |
| Acyl CoA (R) | GGTTCCTTTGACCTGTTTGT | 57 | 45 |
| Acyl CoA-Siderophore (F) | ACAAACAGGTCAAAGGAACC | 57 | 45 |
| Acyl CoA-Siderophore (R) | TATGCAGAGAGGTACGGATG | 57 | 50 |
| Siderophore (F) | CAACTCTCGCTCTCAACTG | 57 | 53 |
| Siderophore (R) | CCAATACGCATTCCACCCA | 57 | 53 |
| Siderophore-Hypothetical (F) | GAGAGAACGAGAAGGTTGTG | 57 | 50 |
| Siderophore-Hypothetical (R) | GACTTGGTGGTAGGGTAACT | 57 | 50 |
| Hypothetical (F) | TCTTAGTTACCCTACCACCA | 55 | 45 |
| Hypothetical (R) | CTTTGATTCGCCCTGCTA | 54 | 50 |

B. Primers for individual genes and inter-regions in the *Ceratocystis* multimodular group

| Primer | Primer sequence (5'→3') | Annealing temperature (⸰C) | GC content (%) |
| --- | --- | --- | --- |
| Hypothetical (F) | TAACTGTCTCTCGGGTTTCC | 58 | 50 |
| Hypothetical (R) | AATTCGAGAGATTGGGGAAG | 57 | 45 |
| Hypothetical-NRPS (F) | TATGCTTCTTTGACCGTACT | 55 | 40 |
| Hypothetical-NRPS (R) | AGGAACATGGAAGAAATGGT | 57 | 40 |
| NRPS (F) | ATTTATCTTATCCCGCACAC | 55 | 40 |
| NRPS (R) | CTTCCAACCAGCAACCGA | 58 | 56 |
| NRPS-Oxygenase (F) | GGGAAAAGGCAGTGAGAGAA | 58 | 50 |
| NRPS-Oxygenase (R) | CCGTCGGAAGCACTATCA | 57 | 56 |
| Oxygenase (F) | TCAGCTCGCCTGTCACTTA | 56 | 53 |
| Oxygenase (R) | CATCTACAATCTCGCCCGA | 57 | 53 |
| Oxygenase-Aspergillopepsin (F) | GGGCGAGATTGTAGATGCT | 58 | 53 |
| Oxygenase-Aspergillopepsin (R) | TGGCGTAGTCGTGTATCTG | 57 | 53 |
| Aspergillopepsin (F) | ACGACTACGCCATGAATATA | 55 | 40 |
| Aspergillopepsin (R) | GTTTGATTATAGCTACGGCT | 54 | 40 |

C. Primers for individual genes in the *Bretziella* monomodular group

| Primer | Primer sequence (5'→3') | Annealing temperature (⸰C) | GC content (%) |
| --- | --- | --- | --- |
| NRPS (F) | GATGACAACTACGGGAGCA | 56 | 53 |
| NRPS (R) | GTCTCCATCTCCACCATCC | 57 | 58 |
| NRPS-Acyl CoA (F) | GTCTCCATCTCCACCATCC | 57 | 47 |
| NRPS-Acyl CoA (R) | TCTTTTGGACTTTGGTGGC | 56 | 47 |
| Acyl CoA (F) | CCAAAGTCCAAAAGATGTCC | 56 | 45 |
| Acyl CoA (R) | ACCCATTCACGAATCTCATC | 57 | 45 |
| Acyl CoA-Siderophore (F) | GAAGATGATGCCTAGTGCTA | 55 | 45 |
| Acyl CoA-Siderophore (R) | TCCCGCGATCTAACCTTT | 56 | 50 |
| Siderophore (F) | CAAAGGTTAGATCGCGGG | 57 | 56 |
| Siderophore (R) | ACCGCAATACTTCCCACA | 58 | 50 |

D. Primers for individual genes in the *Bretziella* multimodular group

| Primer | Primer sequence (5'→3') | Annealing temperature (⸰C) | GC content (%) |
| --- | --- | --- | --- |
| NRPS (F) | ACCCTAACCCTAACCCTAAC | 57 | 50 |
| NRPS (R) | TTTCCAGAAAAACGCCAAAC | 57 | 40 |
| NRPS-Oxygenase (F) | CTGTTTGGCGTTTTTCTGGA | 58 | 45 |
| NRPS-Oxygenase (R) | GAAAGCAATGGGAGAGTTGA | 58 | 45 |
| Oxygenase (F) | GATATCAACTCTCCCATTGC | 55 | 45 |
| Oxygenase (R) | CACTAGAACGAATAGCAAGA | 55 | 40 |
| Oxygenase-Glucanase D (F) | CCTCCTTTCTGTTCTTGCTA | 56 | 45 |
| Oxygenase-Glucanase D (R) | TACGGCTACTCCCAAGAC | 56 | 56 |
| Glucanase D (F) | GTCTTGGGAGTAGCCGTA | 55 | 56 |
| Glucanase D (R) | TACTGCTACCCTCATGGC | 56 | 56 |
| NRPS (F) | ACCCTAACCCTAACCCTAAC | 57 | 50 |
| NRPS (R) | TTTCCAGAAAAACGCCAAAC | 57 | 40 |
| NRPS-Oxygenase (F) | CTGTTTGGCGTTTTTCTGGA | 58 | 45 |
| NRPS-Oxygenase (R) | GAAAGCAATGGGAGAGTTGA | 58 | 45 |

E. Primers for individual genes in the *Huntiella* monomodular group

| Primer | Primer sequence (5'→3') | Annealing temperature (⸰C) | GC content (%) |
| --- | --- | --- | --- |
| NRPS (F) | CTAAGCAATTCTCGCACG | 58 | 50 |
| NRPS (R) | GACTCGTCTGCCTCTTTC | 57 | 56 |
| NRPS-Acyl CoA (F) | GCGAAGCTGAACCATTTT | 55 | 44 |
| NRPS-Acyl CoA (R) | GAAGAATGGAGAGAGCCG | 57 | 56 |
| Acyl CoA (F) | ATCGATACTCCAAACCCC | 55 | 50 |
| Acyl CoA (R) | AAATCACAACCCCAGGCA | 56 | 50 |
| Acyl CoA-Siderophore (F) | GCAAGCGAACAAATATCG | 55 | 44 |
| Acyl CoA-Siderophore (R) | GGAAAGAGTGGTAGCTGG | 55 | 56 |
| Siderophore (F) | CCCAGCTACCACTCTTTC | 56 | 56 |
| Siderophore (R) | TACAGCTTAGCCACCAGA | 54 | 50 |
| Siderophore-Hypothetical (F) | TCTGGTGGCTAAGCTGTA | 55 | 50 |
| Siderophore-Hypothetical (R) | AAGGAGGCTGAGAAAAAG | 55 | 44 |
| Hypothetical (F) | CTACTTTTTCTCAGCCTC | 53 | 44 |
| Hypothetical (R) | TCTTGGATTTGACGAGTG | 53 | 44 |

F. Primers for individual genes in the *Huntiella* multimodular group

| Primer | Primer sequence (5'→3') | Annealing temperature (⸰C) | GC content (%) |
| --- | --- | --- | --- |
| NRPS (F) | CACCAATCACCAATTTCC | 55 | 44 |
| NRPS (R) | AACAAGAAAGCAACGTGC | 57 | 44 |
| NRPS-Oxygenase (F) | ATTGTGAAAGCCTAGTGC | 55 | 44 |
| NRPS-Oxygenase (R) | GAATGAGTGGGTAGTGGG | 56 | 56 |
| Oxygenase (F) | TCACCTTGACCTTGACAT | 55 | 44 |
| Oxygenase (R) | TAGTTCTTGAAGACGGCA | 54 | 44 |
| Oxygenase-Hypothetical (F) | GACACTCTGCTTTCCGTT | 56 | 50 |
| Oxygenase-Hypothetical (R) | TGTTGTCTTTGACGGTGG | 58 | 50 |
| Hypothetical (F) | GGAAGCCCAACCAATAGT | 57 | 50 |
| Hypothetical (R) | TAACTCCCTTCTCCTGGC | 57 | 56 |
| Hypothetical-RNA Pol subunit 7 (F) | AGGGAGTTAAGAGAAGGG | 54 | 50 |
| Hypothetical-RNA Pol subunit 7 (R) | GGAAGTTGACCAGCAAGA | 54 | 50 |
| RNA Pol subunit 7 (F) | CAAGACCCCAACCAAGGA | 57 | 56 |
| RNA Pol subunit 7 (R) | CCACTGGCAAGCTTCAAA | 56 | 50 |

G. Primers for individual genes in the *Davidsoniella* monomodular group

| Primer | Primer sequence (5'→3') | Annealing temperature (⸰C) | GC content (%) |
| --- | --- | --- | --- |
| NRPS (F) | CTTGAACTCCGCCAACTC | 57 | 56 |
| NRPS (R) | CGATCTCGGTTGCGTCTA | 56 | 56 |
| NRPS-Acyl CoA (F) | CCATGGTGAAAGATTGCT | 54 | 44 |
| NRPS-Acyl CoA (R) | TAGACATTTTCTGGCGCT | 55 | 44 |
| Acyl CoA (F) | TCAGCGCCAGAAAATGTC | 57 | 50 |
| Acyl CoA (R) | GTAGGCGTGTTCTTCGTA | 56 | 50 |
| Acyl CoA-Siderophore (F) | GCTACCCCTTCTATGTCTTC | 56 | 50 |
| Acyl CoA-Siderophore (R) | GTATGGGTGAGGAAGCACAA | 57 | 50 |
| Siderophore (F) | TCCCGCTCAGCAACTATC | 57 | 56 |
| Siderophore (R) | TGCGCATACCACCTATAACC | 58 | 50 |
| Siderophore-Hypothetical (F) | TGTAAGGTAGTTAGGGCGTG | 57 | 50 |
| Siderophore-Hypothetical (R) | TGCGAAAAAGGAGGACTCTA | 57 | 45 |
| Hypothetical (F) | TGCGAAAAAGGAGGACTCTA | 55 | 45 |
| Hypothetical (R) | CTTTGATTCGCCCTGCTA | 54 | 50 |

H. Primers for individual genes in the *Davidsoniella* multimodular group

| Primer | Primer sequence (5'→3') | Annealing temperature (⸰C) | GC content (%) |
| --- | --- | --- | --- |
| Hypothetical (F) | TCTGATCACCCCATACCA | 54 | 50 |
| Hypothetical (R) | CTTTCAGCCACCCCATTA | 54 | 50 |
| Hypothetical-NRPS (F) | TAGTAATGGGGTGGCTGAAA | 55 | 45 |
| Hypothetical-NRPS (R) | GAAAAATTGTTGCTGCCCTC | 57 | 45 |
| NRPS (F) | TCTTCTGCCCAATCCTCATC | 57 | 50 |
| NRPS (R) | TCTTCCTACCAGACACCAAA | 56 | 45 |
| NRPS-Oxygenase (F) | TTTGGTGTCTGGTAGGAAGA | 54 | 45 |
| NRPS-Oxygenase (R) | GTCAATATGGGGAGACAT | 52 | 42 |
| Oxygenase (F) | ATGTCTCCCCATATTGACG | 57 | 47 |
| Oxygenase (R) | ACTGCATCAACAATCTCAC | 56 | 42 |
| Oxygenase-Hypothetical (F) | GTGAGATTGTTGATGCAGTT | 56 | 40 |
| Oxygenase-Hypothetical (R) | GTCTGCCTTTGTCGTCTTC | 57 | 53 |
| Hypothetical (F) | TCGAAGACGACAAAGGCAGA | 57 | 50 |
| Hypothetical (R) | CTCTTTTTGTTGCAGCTCTG | 58 | 45 |

I. Primers for individual genes in the *Thielaviopsis* monomodular group

| Primer | Primer sequence (5'→3') | Annealing temperature (⸰C) | GC content (%) |
| --- | --- | --- | --- |
| Hypothetical (F) | CGGGCACAGGAAACAATA | 57 | 50 |
| Hypothetical (R) | ACAACCTCTTCAATCAGC | 55 | 44 |
| Hypothetical-NRPS (F) | GAAGCTGATTGAAGAGGT | 55 | 44 |
| Hypothetical-NRPS (R) | GCTGGAAATGATGCGGAA | 55 | 50 |
| NRPS (F) | TGCCTTCAGCTCTGCCAA | 58 | 56 |
| NRPS (R) | ACTCAAAAATGGTCCAGC | 55 | 44 |
| NRPS-Acyl CoA (F) | GCTGGACCATTTTTGAGTAA | 55 | 44 |
| NRPS-Acyl CoA (R) | TCGATGCGGGTCTGAGTA | 57 | 56 |
| Acyl CoA (F) | ATGCTTTCCATTCTCCAC | 55 | 44 |
| Acyl CoA (R) | TACAGCTTCGATTTGGGC | 57 | 50 |
| Acyl CoA-Siderophore (F) | GCCCAAATCGAAGCTGTA | 56 | 50 |
| Acyl CoA-Siderophore (R) | GCGAATGGTGTTTGACAT | 56 | 44 |
| Siderophore (F) | ATGTCAAACACCATTCGC | 56 | 44 |
| Siderophore (R) | TTACTCGCCATAAGCCTT | 55 | 44 |
| Siderophore-Aerobactin (F) | CGCAGTGCAAAAGAGCTA | 56 | 50 |
| Siderophore-Aerobactin (R) | CAGGCACGTCAAGAGAGA | 55 | 56 |
| Aerobactin (F) | GTCTCTCTTGACGTGCCT | 57 | 56 |
| Aerobactin (R) | CATGCCTCCAACAACCTT | 57 | 50 |

J. Primers for individual genes in the *Thielaviopsis* multimodular group

| Primer | Primer sequence (5'→3') | Annealing temperature (⸰C) | GC content (%) |
| --- | --- | --- | --- |
| NRPS (F) | TTAAACCCACTAACACGG | 53 | 44 |
| NRPS (R) | GGTCCCAATCTTTTACAC | 52 | 44 |
| NRPS-Oxygenase (F) | GTTGAGAATGGCCAGAGC | 56 | 56 |
| NRPS-Oxygenase (R) | GCAGGGAGAACATCGTCA | 58 | 56 |
| Oxygenase (F) | GATCATCTGGACCTCGAC | 55 | 56 |
| Oxygenase (R) | GAGCGGATGGAAAGAACC | 57 | 56 |
| Oxygenase-Endothiapepsin (F) | CACTCTCCTGTCGGTTCT | 58 | 56 |
| Oxygenase-Endothiapepsin (R) | CAACGAAGCCTCTGACAG | 58 | 56 |
| Endothiapepsin (F) | CAACGAAGCCTCTGACAG | 57 | 44 |
| Endothiapepsin (R) | TTCCTTATGGCGGCTCTT | 57 | 50 |
| Endothiapepsin-Hypothetical (F) | GTTGACAGAGGGAGACAT | 55 | 50 |
| Endothiapepsin-Hypothetical (R) | TGTTGCTGGCGTAGAAGA | 56 | 50 |
| Hypothetical (F) | CTCTTCTACGCCAGCAAC | 57 | 56 |
| Hypothetical (R) | TTGACTCCTCCTCCCCTT | 58 | 56 |

K. Primers for individual genes in the *Endoconidiophora* monomodular group

| Primer | Primer sequence (5'→3') | Annealing temperature (⸰C) | GC content (%) |
| --- | --- | --- | --- |
| Hypothetical (F) | AAATAGTGGTGTATCGGG | 54 | 44 |
| Hypothetical (R) | CTTCTGGATGCCTCTTCT | 55 | 50 |
| Hypothetical-NRPS (F) | GAAGAGGCATCCAGAAGG | 57 | 56 |
| Hypothetical-NRPS (R) | GATTTGATACGCCGCGAG | 58 | 56 |
| NRPS (F) | ATGCGCACCTTAAACTCT | 57 | 44 |
| NRPS (R) | GCAACTTTGGGGGTTCTG | 57 | 56 |
| NRPS-Acyl CoA (F) | CAGAACCCCCAAAGTTGC | 57 | 56 |
| NRPS-Acyl CoA (R) | AGACATTTTCTGGCGCTG | 58 | 50 |
| Acyl CoA (F) | CAGCGCCAGAAAATGTCT | 57 | 50 |
| Acyl CoA (R) | TTAACCAGGTCGTTGCCC | 58 | 56 |
| Acyl CoA-Siderophore (F) | GGTTAAGAAGACCACGCT | 57 | 50 |
| Acyl CoA-Siderophore (R) | GGCAGGAGAAGCCATTAT | 56 | 50 |
| Siderophore (F) | TAATGGCTTCTCCTGCCC | 57 | 56 |
| Siderophore (R) | ACCTTTTCATTGTCGCCT | 57 | 44 |

L. Primers for individual genes in the *Endoconidiophora* multimodular group

| Primer | Primer sequence (5'→3') | Annealing temperature (⸰C) | GC content (%) |
| --- | --- | --- | --- |
| RNA Pol II subunit 7 (F) | GGATGACTCAGGGCAGAA | 57 | 56 |
| RNA Pol II subunit 7 (R) | TCAAGGTCACGTTTTTCCAA | 56 | 40 |
| RNA Pol II subunit 7-Endothiapepsin (F) | CGAGACGCACTATTGGAA | 56 | 50 |
| RNA Pol II subunit 7-Endothiapepsin (R) | ACGAGAAGGAGGTTTATG | 54 | 44 |
| Endothiapepsin (F) | CGCCTTTCATAAACCTCC | 55 | 50 |
| Endothiapepsin (R) | GCCGTCGAAAACCACAAA | 56 | 50 |
| Endothiapepsin-Oxygenase (F) | GTAAACTGGAAAGAAGGG | 53 | 44 |
| Endothiapepsin-Oxygenase (R) | GCTCCGGTGAAATTGTTG | 55 | 50 |
| Oxygenase (F) | GCATCAACAATTTCACCG | 54 | 44 |
| Oxygenase (R) | TGTCTCCCCATATTGACG | 54 | 50 |
| Oxygenase-NRPS (F) | CGTCAATATGGGGAGACA | 57 | 50 |
| Oxygenase-NRPS (R) | TGGCCCATGGATAGTGGA | 56 | 56 |
| NRPS (F) | CACTATCCATGGGCCAAC | 57 | 56 |
| NRPS (R) | TCTTCATCGGCTACCTCT | 55 | 50 |
