# Supplementary material for: Distribution and Evolution of Nonribosomal Peptide Synthetase Gene Clusters in the Ceratocystidaceae
Source: Genes (Basel). 2019 Apr 30;10(5):328. doi: 10.3390/genes10050328 (PMC6563098; doi:10.3390/genes10050328)
Supplement: Supplementary file 1 [file genes-10-00328-s001.zip › Supplementary Files/Supplementary file S5 Feb 2019.docx]

**SUPPLEMENTARY FILE S-5.**

*(Sayari et al - Ceratocystidaceae Nonribosomal peptide synthetase gene clusters)*

Top 10 BLASTp hits for each NRPS sequences. BLASTp search was done against the non-redundant protein sequences in the National Centre for Biotechnology Information database (NCBI; http://blast.ncbi.nlm.nih.gov/), and the top ten hits are indicated with species where they are found, E-value, percent sequence identity and coverage.

A) *Ceratocystis* monomodular NRPS

| *Ceratocystis* |  |  |  |  |
| --- | --- | --- | --- | --- |
| *Ceratocystis* monomodular NRPS | Species of BLAST hit | % coverage | % identity | E-value |
| NRPS | *Colletotrichum gloeosporioides* | 88 | 51 | 0 |
| NRPS | *Colletotrichum higginsianum* | 87 | 50 | 0 |
| NRPS | *Colletotrichum higginsianum* | 87 | 51 | 0 |
| NRPS | *Colletotrichum graminicola* | 86 | 51 | 0 |
| NRPS | *Colletotrichum fioriniae* | 86 | 51 | 0 |
| NRPS | *Verticillium dahliae* | 90 | 49 | 0 |
| NRPS | *Verticillium dahliae* | 86 | 51 | 0 |
| NRPS | *Verticillium dahliae* | 86 | 51 | 0 |
| NRPS | *Metarhizium majus* | 83 | 51 | 0 |
| NRPS | *Metarhizium robertsii* | 83 | 51 | 0 |

B) *Ceratocystis* mulitmodular-modular NRPS

| *Ceratocystis* |  |  |  |  |
| --- | --- | --- | --- | --- |
| *Ceratocystis* mulitmodular-modular NRPS | Species of BLAST hit | % coverage | % identity | E-value |
| NRPS | *Scedosporium apiospermum* | 99 | 51 | 0 |
| NRPS | *Colletotrichum higginsianum* | 99 | 49 | 0 |
| NRPS | *Colletotrichum graminicola* | 99 | 49 | 0 |
| NRPS | *Nectria haematococca* | 99 | 49 | 0 |
| NRPS | *Colletotrichum gloeosporioides* | 99 | 49 | 0 |
| NRPS | *Fusarium graminearum* | 99 | 48 | 0 |
| NRPS | *Trichoderma virens* | 99 | 48 | 0 |
| NRPS | *Fusarium pseudograminearum* | 99 | 48 | 0 |
| NRPS | *Metarhizium robertsii* | 99 | 48 | 0 |
| NRPS | *Metarhizium brunneum* | 99 | 48 | 0 |

C) *Huntiella* monomodular NRPS

| *Huntiella* |  |  |  |  |
| --- | --- | --- | --- | --- |
| *Huntiella* monomodular NRPS | Species of BLAST hit | % coverage | % identity | E-value |
| NRPS | *Colletotrichum gloeosporioides* | 90 | 54 | 0 |
| NRPS | *Colletotrichum higginsianum* | 87 | 55 | 0 |
| NRPS | *Colletotrichum higginsianum* | 86 | 55 | 0 |
| NRPS | *Verticillium dahliae* | 92 | 52 | 0 |
| NRPS | *Verticillium dahliae* | 83 | 56 | 0 |
| NRPS | *Verticillium dahliae* | 87 | 55 | 0 |
| NRPS | *Colletotrichum graminicola* | 88 | 54 | 0 |
| NRPS | *Colletotrichum fioriniae* | 83 | 54 | 0 |
| NRPS | *Metarhizium majus* | 83 | 54 | 0 |
| NRPS | *Metarhizium brunneum* | 83 | 54 | 0 |

D) *Huntiella* mulitmodular-modular NRPS

| *Huntiella* |  |  |  |  |
| --- | --- | --- | --- | --- |
| *Huntiella* mulitmodular-modular NRPS | Species of BLAST hit | % coverage | % identity | E-value |
| NRPS | *Nectria haematococca* | 99 | 47 | 0 |
| NRPS | *Trichoderma reesei* | 98 | 47 | 0 |
| NRPS | *Trichoderma virens* | 99 | 47 | 0 |
| NRPS | *Trichoderma reesei* | 98 | 47 | 0 |
| NRPS | *Fusarium graminearum* | 99 | 47 | 0 |
| NRPS | *Thielavia terrestris* | 99 | 47 | 0 |
| NRPS | *Colletotrichum higginsianum* | 99 | 47 | 0 |
| NRPS | *Colletotrichum gloeosporioides* | 99 | 47 | 0 |
| NRPS | *Fusarium pseudograminearum* | 99 | 47 | 0 |
| NRPS | *Trichoderma atroviride* | 99 | 47 | 0 |

E) *Thielaviopsis* monomodular NRPS

| *Thielaviopsis* |  |  |  |  |
| --- | --- | --- | --- | --- |
| *Thielaviopsis* monomodular NRPS | Species of BLAST hit | % coverage | % identity | E-value |
| NRPS | *Colletotrichum gloeosporioides* | 86 | 56 | 0 |
| NRPS | *Colletotrichum higginsianum* | 86 | 56 | 0 |
| NRPS | *Verticillium dahliae* | 92 | 52 | 0 |
| NRPS | *Colletotrichum higginsianum* | 86 | 56 | 0 |
| NRPS | *Verticillium dahliae* | 92 | 52 | 0 |
| NRPS | *Verticillium dahliae* | 92 | 52 | 0 |
| NRPS | *Colletotrichum graminicola* | 86 | 55 | 0 |
| NRPS | *Colletotrichum fioriniae* | 86 | 55 | 0 |
| NRPS | *Metarhizium brunneum* | 83 | 56 | 0 |
| NRPS | *Metarhizium majus* | 83 | 55 | 0 |

F) *Thielaviopsis* mulitmodular-modular NRPS

| *Thielaviopsis* |  |  |  |  |
| --- | --- | --- | --- | --- |
| *Thielaviopsis* mulitmodular-modular NRPS | Species of BLAST hit | % coverage | % identity | E-value |
| NRPS | *Fusarium oxysporum* | 99 | 39 | 0 |
| NRPS | *Colletotrichum higginsianum* | 99 | 50 | 0 |
| NRPS | *Nectria haematococca* | 99 | 50 | 0 |
| NRPS | *Colletotrichum graminicola* | 99 | 50 | 0 |
| NRPS | *Fusarium graminearum* | 99 | 50 | 0 |
| NRPS | *Colletotrichum gloeosporioides* | 99 | 50 | 0 |
| NRPS | *Trichoderma virens* | 99 | 49 | 0 |
| NRPS | *Fusarium pseudograminearum* | 99 | 50 | 0 |
| NRPS | *Trichoderma reesei* | 99 | 49 | 0 |
| NRPS | *Trichoderma reesei* | 99 | 49 | 0 |

G) *Endoconidiophora* monomodular NRPS

| *Endoconidiophora* |  |  |  |  |
| --- | --- | --- | --- | --- |
| *Endoconidiophora* monomodular NRPS | Species of BLAST hit | % coverage | % identity | E-value |
| NRPS | *Colletotrichum gloeosporioides* | 86 | 56 | 0 |
| NRPS | *Colletotrichum higginsianum* | 86 | 56 | 0 |
| NRPS | *Colletotrichum higginsianum* | 86 | 56 | 0 |
| NRPS | *Verticillium dahliae* | 92 | 52 | 0 |
| NRPS | *Verticillium dahliae* | 86 | 54 | 0 |
| NRPS | *Verticillium dahliae* | 86 | 54 | 0 |
| NRPS | *Colletotrichum graminicola* | 86 | 55 | 0 |
| NRPS | *Colletotrichum fioriniae* | 86 | 55 | 0 |
| NRPS | *Metarhizium majus* | 83 | 54 | 0 |
| NRPS | *Metarhizium brunneum* | 83 | 54 | 0 |

H) *Endoconidiophora* mulitmodularmodular-NRPS

| *Endoconidiophora* |  |  |  |  |
| --- | --- | --- | --- | --- |
| *Endoconidiophora* mulitmodular-modular NRPS | Species of BLAST hit | % coverage | % identity | E-value |
| NRPS | *Scedosporium apiospermum* | 99 | 59 | 0 |
| NRPS | *Nectria haematococca* | 99 | 58 | 0 |
| NRPS | *Trichoderma virens* | 99 | 58 | 0 |
| NRPS | *Trichoderma raesie* | 99 | 58 | 0 |
| NRPS | *Fusarium graminearum* | 99 | 58 | 0 |
| NRPS | *Fusarium pseudograminearum* | 99 | 58 | 0 |
| NRPS | *Trichoderma reesei* | 97 | 58 | 0 |
| NRPS | *Colletotrichum graminicola* | 98 | 58 | 0 |
| NRPS | *Metarhizium robertsii* | 98 | 57 | 0 |
| NRPS | *Metarhizium brunneum* | 99 | 57 | 0 |

I) *Davidsoniella* monomodular NRPS

| *Davidsoniella* |  |  |  |  |
| --- | --- | --- | --- | --- |
| *Davidsoniella* monomodular NRPS | Species of BLAST hit | % coverage | % identity | E-value |
| NRPS | *Colletotrichum gloeosporioides* | 85 | 57 | 0 |
| NRPS | *Colletotrichum higginsianum* | 86 | 56 | 0 |
| NRPS | *Colletotrichum higginsianum* | 86 | 56 | 0 |
| NRPS | *Colletotrichum graminicola* | 86 | 56 | 0 |
| NRPS | *Colletotrichum fioriniae* | 87 | 55 | 0 |
| NRPS | *Verticillium dahliae* | 92 | 52 | 0 |
| NRPS | *Verticillium dahliae* | 87 | 54 | 0 |
| NRPS | *Verticillium dahliae* | 87 | 54 | 0 |
| NRPS | *Metarhizium brunneum* | 83 | 55 | 0 |
| NRPS | *Metarhizium majus* | 83 | 54 | 0 |

J) *Davidsoniella* mulitmodular-modular NRPS

| *Davidsoniella* |  |  |  |  |
| --- | --- | --- | --- | --- |
| *Davidsoniella* mulitmodular-modular NRPS | Species of BLAST hit | % coverage | % identity | E-value |
| NRPS | *Nectria haematococca* | 99 | 58 | 0 |
| NRPS | *Scedosporium apiospermum* | 98 | 59 | 0 |
| NRPS | *Trichoderma reesei* | 99 | 58 | 0 |
| NRPS | *Trichoderma virens* | 99 | 59 | 0 |
| NRPS | *Trichoderma reesei* | 99 | 58 | 0 |
| NRPS | *Fusarium pseudograminearum* | 99 | 58 | 0 |
| NRPS | *Fusarium graminearum* | 99 | 58 | 0 |
| NRPS | *Colletotrichum higginsianum* | 99 | 58 | 0 |
| NRPS | *Metarhizium robertsii* | 99 | 57 | 0 |
| NRPS | *Metarhizium acridum* | 99 | 57 | 0 |

N) *Ambrosiella xylerobi* PCDO01000001, NRPS

| *Ambrosiella xylerobi* |  |  |  |  |
| --- | --- | --- | --- | --- |
| *Knoxdaviesia* mulitmodular-modular NRPS | Species of BLAST hit | % coverage | % identity | E-value |
| NRPS | *Colletotrichum gloeosporioides* | 86 | 55 | 0 |
| NRPS | *Verticillium dahliae* | 91 | 51 | 0 |
| NRPS | *Colletotrichium obiculare* | 86 | 55 | 0 |
| NRPS | *Colletotrichum gloeosporioides* | 86 | 55 | 0 |
| NRPS | *Colletotrichum gloeosporioides* | 86 | 55 | 0 |
| NRPS | *Verticillium dahliae* | 91 | 51 | 0 |
| NRPS | *Verticillium dahliae* | 91 | 51 | 0 |
| NRPS | *Verticillium dahliae* | 91 | 51 | 0 |
| NRPS | *Colletotrichum chlorophyti* | 86 | 54 | 0 |
| NRPS | *Verticillium longisporum* | 91 | 51 | 0 |

O) *Ambrosiella xylerobi* PCDO01000004, NRPS

| *Ambrosiella xylerobi* |  |  |  |  |
| --- | --- | --- | --- | --- |
| *Knoxdaviesia* mulitmodular-modular NRPS | Species of BLAST hit | % coverage | % identity | E-value |
| NRPS | *Scedosporium apiospermum* | 99 | 38 | 0 |
| NRPS | *Scedosporium apiospermum* | 99 | 38 | 0 |
| NRPS | *Neonectria ditissima* | 99 | 38 | 0 |
| NRPS | *Trichoderma citrinoviride* | 99 | 38 | 0 |
| NRPS | *Nectria haematococca* | 99 | 38 | 0 |
| NRPS | *Colletotrichum tofieldia* | 99 | 38 | 0 |
| NRPS | *Purpureocillium lilacinum* | 99 | 38 | 0 |
| NRPS | *Colletotrichum sublinecola* | 99 | 37 | 0 |
| NRPS | *Tolypocladium paradoxum* | 99 | 38 | 0 |
| NRPS | *Colletotrichum incanum* | 99 | 38 | 0 |

P) *Davidsoniella australis* contig0000143 monomodular NRPS

| *Davidsoniella australis* |  |  |  |  |
| --- | --- | --- | --- | --- |
| *Davidsoniella* monomodular NRPS | Species of BLAST hit | % coverage | % identity | E-value |
| NRPS | *Metarhizium album* | 99 | 38 | 0 |
| NRPS | *Nectria haematococca* | 99 | 39 | 0 |
| NRPS | *Fusarium avenaceum* | 99 | 39 | 0 |
| NRPS | *Pochonia chlamydosporia* | 99 | 38 | 0 |
| NRPS | *Hirsutella minnesotensis* | 99 | 38 | 0 |
| NRPS | *Metarhizium album* | 99 | 38 | 0 |
| NRPS | *Cordyceps confragosa* | 98 | 38 | 0 |
| NRPS | *Cordyceps confragosa* | 99 | 37 | 0 |
| NRPS | *Ophiocordyceps camponoti-rufipedis* | 99 | 38 | 0 |
| NRPS | *Scedosporium apiospermum* | 97 | 39 | 0 |

Q) *Davidsoniella australis* contig0000001 mulitmodular-modular NRPS

| *Davidsoniella australis* |  |  |  |  |
| --- | --- | --- | --- | --- |
| *Davidsoniella* mulitmodular-modular NRPS | Species of BLAST hit | % coverage | % identity | E-value |
| NRPS | *Colletotrichum orbiculare* | 86 | 56 | 0 |
| NRPS | *Colletotrichum higginsianum* | 86 | 56 | 0 |
| NRPS | *Colletotrichum gloeosporioides* | 86 | 56 | 0 |
| NRPS | *Colletotrichum gloeosporioides* | 86 | 56 | 0 |
| NRPS | *Colletotrichum orbiculare* | 86 | 56 | 0 |
| NRPS | *Colletotrichum higginsianum* | 86 | 56 | 0 |
| NRPS | *Colletotrichum higginsianum* | 86 | 56 | 0 |
| NRPS | *Colletotricium chlorophyti* | 86 | 55 | 0 |
| NRPS | *Colletotrichum tofieldia* | 86 | 55 | 0 |
| NRPS | *Colletotricium incanum* | 86 | 55 | 0 |

R) *Davidsoniella neocaledoniae* contig000006 monomodular NRPS

| *Davidsoniella neocaledoniae* |  |  |  |  |
| --- | --- | --- | --- | --- |
| *Davidsoniella* monomodular NRPS | Species of BLAST hit | % coverage | % identity | E-value |
| NRPS | *Fusarium oxysporum* | 99 | 40 | 0 |
| NRPS | *Fusarium oxysporum* | 99 | 40 | 0 |
| NRPS | *Fusarium oxysporum* | 99 | 40 | 0 |
| NRPS | *Fusarium oxysporum* | 99 | 40 | 0 |
| NRPS | *Fusarium oxysporum* | 99 | 40 | 0 |
| NRPS | *Fusarium oxysporum* | 99 | 40 | 0 |
| NRPS | *Fusarium oxysporum* | 99 | 40 | 0 |
| NRPS | *Fusarium oxysporum* | 99 | 40 | 0 |
| NRPS | *Fusarium oxysporum* | 99 | 39 | 0 |
| NRPS | *Fusarium oxysporum* | 99 | 40 | 0 |

S) *Davidsoniella neocaledoniae* contig00001989 mulitmodular-modular NRPS

| *Davidsoniella neocaledoniae* |  |  |  |  |
| --- | --- | --- | --- | --- |
| *Davidsoniella* mulitmodular-modular NRPS | Species of BLAST hit | % coverage | % identity | E-value |
| NRPS | *Thelaviopsis punctulata* | 98 | 75 | 0 |
| NRPS | *Cerarocystis platani* | 97 | 62 | 0 |
| NRPS | *Ceratocystis fimbriata* | 98 | 56 | 0 |
| NRPS | *Colletotrichum gloeosporioides* | 94 | 51 | 0 |
| NRPS | *Colletotrichum gloeosporioides* | 94 | 51 | 0 |
| NRPS | *Colletotrichum obiculare* | 96 | 50 | 0 |
| NRPS | *Colletotrichum higginsianum* | 95 | 50 | 0 |
| NRPS | *Colletotrichum higginsianum* | 94 | 51 | 0 |
| NRPS | *Colletotrichum tofieldia* | 95 | 50 | 0 |
| NRPS | *Colletotrichum chlorophyti* | 95 | 50 | 0 |

T) *Berkeleyomyces basicola* Contig 12, monomodular NRPS

| *Thielaviopsis basicola* |  |  |  |  |
| --- | --- | --- | --- | --- |
| *Thielaviopsis basicola* monomodular NRPS | Species of BLAST hit | % coverage | % identity | E-value |
| NRPS | *Scedosporium apiospermum* | 99 | 41 | 0 |
| NRPS | *Lomentospora prolificans* | 99 | 41 | 0 |
| NRPS | *Neonectria ditissima* | 99 | 41 | 0 |
| NRPS | *Nectria haematococca* | 99 | 41 | 0 |
| NRPS | *Colletotrichum sublineola* | 99 | 40 | 0 |
| NRPS | *Colletotrichum tofieldia* | 99 | 40 | 0 |
| NRPS | *Trichoderma reesi* | 99 | 40 | 0 |
| NRPS | *Fusarium fujikuroi* | 99 | 40 | 0 |
| NRPS | *Colletotrichum incanum* | 99 | 40 | 0 |
| NRPS | *Fusarium fujikuroi* | 99 | 40 | 0 |

T) *Berkeleyomyces basicola* Contig 8, mulitmodular-modular NRPS

| *Thielaviopsis basicola* |  |  |  |  |
| --- | --- | --- | --- | --- |
| *Thielaviopsis basicola* monomodular NRPS | Species of BLAST hit | % coverage | % identity | E-value |
| NRPS | *Colletotrichum gloeosporioides* | 86 | 54 | 0 |
| NRPS | *Colletotrichum gloeosporioides* | 86 | 54 | 0 |
| NRPS | *Colletotrichum tofieldia* | 86 | 54 | 0 |
| NRPS | *Colletotrichum chlorophyti* | 86 | 54 | 0 |
| NRPS | *Colletotrichum orbiculare* | 86 | 54 | 0 |
| NRPS | *Colletotrichum incanum* | 86 | 54 | 0 |
| NRPS | *Colletotrichum incanum* | 86 | 54 | 0 |
| NRPS | *Colletotrichum higginsanum* | 86 | 54 | 0 |
| NRPS | *Colletotrichum higginsianum* | 86 | 54 | 0 |
| NRPS | *Colletotrichum sublineola* | 86 | 54 | 0 |
