# Supplementary material for: Distribution and Evolution of Nonribosomal Peptide Synthetase Gene Clusters in the Ceratocystidaceae
Source: Genes (Basel). 2019 Apr 30;10(5):328. doi: 10.3390/genes10050328 (PMC6563098; doi:10.3390/genes10050328)
Supplement: Supplementary file 1 [file genes-10-00328-s001.zip › Supplementary Files/Supplementary file S1 FEb 2019.docx]

**SUPPLEMENTARY FILE S1.**

*(Sayari et al - Ceratocystidaceae Nonribosomal peptide synthetase gene clusters)*

Genome sequence information for the 16 *Sordariomycetes* representative included in this study. Secondary Metabolite Unique Regions Finder (SMURF; www.jcvi.org/smurf/; (Khaldi et al. 2010) was used to predict NRPS genes from the *Sordariomycetes* genomes available from Joint Genome Institute (JGI; https://jgi.doe.gov/our-science/science-programs/fungal-genomics/) and National Centre for Biotechnology Information (NCBI; <http://blast.ncbi.nlm.nih.gov/>).

| **Species** | **GenBank Accession number** | **References** |
| --- | --- | --- |
| *Ophiostoma novo-ulmi* | AORF01000001 | Blanco-Ulate *et al.,* 2013 |
| *Melanospora tiffanyae* | SRX3340958 | https://genome.jgi.doe.gov/Melti1/Melti1.home.html |
| *Sordaria macrospora* | CABT00000000 | Nowrousian *et al.,* 2010 |
| *Verticillium tricorpus* | JPET00000000 | Seidl *et al.,* 2015 |
| *Nectria haematococca* | NZ_ACJF00000000 | Coleman *et al.,* 2009 |
| *Cordyceps militaris* | AEVU00000000 | Zheng *et al.,* 2012 |
| *Claviceps purpurea* | CAGA00000000 | Schardl *et al.,* 2013 |
| *Colletotrichum gloeosporioides* | QFRH00000000 | Masel *et al.,* 1990 |
| *Stachybotrys chartarum* | LDEE00000000 | Semeiks *et al.,* 2014 |
| *Xylaria hypoxylon* | JWIU00000000 | http://genome.jgi.doe.gov/Xylhyp1/Xylhyp1.home.html |
| *Eutypa lata* | AORF00000000 | Blanco-Ulate et al., 2013 |
| *Magnaporthe oryzae* | AACU00000000 | Dong *et al.,* 2015 |
| *Diaporthe longicolla* | JUJX00000000 | Li *et al.,* 2015 |
| *Chaetomium globosum* | AAFU00000000 | Cuomo *et al.,* 2015 |
| *Microascus trigonosporus* | PRJNA196081 | https://genome.jgi.doe.gov/Mictr1/Mictr1.home.html |
