# Supplementary material for: Distribution and Evolution of Nonribosomal Peptide Synthetase Gene Clusters in the Ceratocystidaceae
Source: Genes (Basel). 2019 Apr 30;10(5):328. doi: 10.3390/genes10050328 (PMC6563098; doi:10.3390/genes10050328)
Supplement: Supplementary file 1 [file genes-10-00328-s001.zip › Supplementary Files/Supplementary File S4 Feb 2019.docx]

**SUPPLEMENTARY FILE S4.**

*(Sayari et al - Ceratocystidaceae Nonribosomal peptide synthetase gene clusters)*

The tables below show the putative Nonribosomal Peptide Synthetase (NRPS) gene clusters predicted by SMURF (Secondary Metabolite Unique Regions Finder) (Khaldi et al., 2010; Medema et al. 2011).

| *Ceratocystis adiposa* multimodular NRPS cluster - Contig 175 (LXGU00000175) | | | | | | | | | | |
| --- | --- | --- | --- | --- | --- | --- | --- | --- | --- | --- |
| Backbone_gene_id | **Gene_id** | **Gene positions** | **Contig** | **Gene order** | **5'-3’ end** | **Gene distance** | | **Domain score** | **Annotated gene function** | |
| g26 | g24 | 2 | 175 | 7 | 664-1427 | 1956 | | 0 | Glutathione transferase | |
| g26 | g23 | 3 | 175 | 6 | 6275-7575 | 293 | | 0 | Transposase | |
| g26 | g22 | 4 | 175 | 5 | 8168-22785 | 0 | | 1 | Hydroxymate type ferrichrome siderophore peptide synthase | |
| g26 | g21 | 5 | 175 | 4 | 32170-33814 | 1442 | | 1 | L-ornithine N-5 monooxygenase | |
| g26 | g20 | 6 | 175 | 3 | 35185-36432 | 521 | | 0 | Endothiapepsin | |
| g26 | g25 | 10 | 175 | 8 | 39445-40399 | 196 | | 0 | Mediator of RNA polymerase-II transcription subutit 7 | |
| g26 | g19 | 7 | 175 | 2 | 40811-41782 | 327 | |  | Hypothetical | |
| g26 | g20 | 8 | 175 | 1 | 664-1427 | 405 | |  | Glutathione transferase | |
| g26 | g18 | 9 | 175 | 9 | 6275-7575 | 1717 | |  | Transposase | |
|  | | | | | | | | | | |
| *Ceratocystis albifundus* multimodular NRPS cluster - Contig1169 (JSSU000001169) | | | | | | | | | | |
| Backbone_gene_id | **Gene_id** | **Gene positions** | **Contig** | **Gene order** | **5'-3’ end** | **Gene distance** | | **Domain score** | **Annotated gene function** | |
| g31 | g33 | -2 | 1169 | 7 | 470-3248 | 0 | | 1 | Hydroxymate type ferrichrome siderophore peptide synthase | |
| g31 | g32 | -1 | 1169 | 6 | 3503-4522 | 2611 | | 1 | L-ornithine N-5 monooxygenase | |
| g31 | g31 | 0 | 1169 | 5 | 5694-8351 | 3258 | | 1 | Mediator of RNA polymerase-II transcription subutit 7 | |
| g31 | g27 | 6 | 1169 | 1 | 9455-13612 | 348 | | 1 | Hypothetical | |
|  | | | | | | | | | | |
| *Ceratocystis eucalypticola* multimodular NRPS cluster - Contig 206 (LJOA00000206) | | | | | | | | | | |
| Backbone_gene_id | **Gene_id** | **Gene positions** | **Contig** | **Gene order** | **5'-3’ end** | **Gene distance** | **Domain score** | | **Annotated gene function** | |
| g4 | g11 | -7 | 206 | 11 | 7544-8599 | 1446 | 1 | | Hypothetical | |
| g4 | g10 | -6 | 206 | 10 | 9147-9974 | 1463 | 0 | | Mediator of RNA polymerase-II transcription subutit 7 | |
| g4 | g9 | -5 | 206 | 9 | 11685-12962 | 171 | 0 | | Aspergillopepsin | |
| g4 | g8 | -4 | 206 | 8 | 14568-15786 | 610 | 0 | | L-ornithine N-5 monooxygenase | |
| g4 | g7 | -3 | 206 | 7 | 21836-36887 | 607 | 0 | | Hydroxymate type ferrichrome siderophore peptide synthase | |
|  | | | | | | | | | | |
| *Bretziella fagacearum* multimodular NRPS cluster - Contig 227 (MKGJ00000227) | | | | | | | | | | |
| Backbone_gene_id | **Gene_id** | **Gene positions** | **Contig** | **Gene order** | **5'-3’ end** | **Gene distance** | **Domain score** | | **Annotated gene function** | |
| MJMS01000056_g6 | MKGJ00000227_g9 | -3 | 227 | 4 | 1-14960 | 0 | 0 | | Hydroxymate type ferrichrome siderophore peptide synthase | |
| MJMS01000056_g6 | MKGJ00000227_g8 | -2 | 227 | 3 | 21912-23581 | 1834 | 0 | | L-ornithine N-5 monooxygenase | |
| MJMS01000056_g6 | MKGJ00000227_g7 | -1 | 227 | 2 | 24912-26018 | 901 | 0 | | 1,4 glucanase | |
| MJMS01000056_g6 | MKGJ00000227_g6 | 0 | 227 | 1 | 28750-31021 | 1757 | 0 | | Chitinase | |
| MJMS01000056_g6 | MKGJ00000227_g10 1 56 | 1 | 227 | 5 | 35659-37651 | 3335 | 1 | | Hypothetical | |
|  | | | | | | | | | | |
| *Ceratocystis manginecans* multimodular NRPS cluster - Contig 189 ([JJRZ000000189](http://www.ncbi.nlm.nih.gov/nuccore/659897781)) | | | | | | | | | | |
| Gene positions | **Chromosome-Contig** | **Gene_position** | **Contig** | **Gene order** | **5'-3’ end** | **Gene distance Domain score Annotated gene function** | | | | |
| JMSH01000003_g25 | [JJRZ000000189](http://www.ncbi.nlm.nih.gov/nuccore/659897781)_g31 | -6 | 189 | 5 | 24617-39671 | 267 | 1 | | Hydroxymate type ferrichrome siderophore peptide synthase | |
| JMSH01000003_g25 | [JJRZ000000189](http://www.ncbi.nlm.nih.gov/nuccore/659897781)_g30 | -5 | 189 | 4 | 46319-47994 | 1861 | 1 | | L-ornithine N-5 monooxygenase | |
| JMSH01000003_g25 | [JJRZ000000189](http://www.ncbi.nlm.nih.gov/nuccore/659897781)_g29 | -4 | 189 | 3 | 48781-50058 | 274 | 0 | | Aspergillopepsin | |
| JMSH01000003_g25 | [JJRZ000000189](http://www.ncbi.nlm.nih.gov/nuccore/659897781)_g28 | -3 | 189 | 2 | 51768-52595 | 1025 | 0 | | Mediator of RNA polymerase-II transcription subutit 7 | |
| JMSH01000003_g25 | [JJRZ000000189](http://www.ncbi.nlm.nih.gov/nuccore/659897781)_g27 | -2 | 189 | 1 | 53143-54198 | 285 | 0 | | Hypothetical | |
|  | | | | | | | | | | |
| *Ceratocystis fimbriata* multimodular NRPS cluster - Contig 182 ([APWK00000182)](http://www.ncbi.nlm.nih.gov/nuccore/APWK00000000) | | | | | | | | | | |
| Backbone_gene_id | **Gene_id** | **Gene positions** | **Contig** | **Gene order** | **5'-3’ end** | **Gene distance** | **Domain score** | | | **Annotated gene function** |
| LXKZ01000105_g23 | APWK00000182_g21 | 1 | 182 | 7 | 24617-39671 | 473 | 0 | | | Hydroxymate type ferrichrome siderophore peptide synthase |
| LXKZ01000105_g23 | APWK00000182_g20 | 2 | 182 | 6 | 46319-47994 | 699 | 1 | | | L-ornithine N-5 monooxygenase |
| LXKZ01000105_g23 | APWK00000182_g18 | 3 | 182 | 4 | 48781-50058 | 3613 | 0 | | | Aspergillopepsin |
| LXKZ01000105_g23 | APWK00000182_g17 | 4 | 182 | 3 | 51768-52595 | 130 | 1 | | | Mediator of RNA polymerase-II transcription subutit 7 |
| LXKZ01000105_g23 | APWK00000182_g16 | 5 | 182 | 2 | 53143-54198 | 606 | 0 | | | Hypothetical |
|  | | | | | | | | | | |
| *Ceratocystis harringtonii* multimodular NRPS cluster - Contig 378 (MKGM0000378) | | | | | | | | | | |
| Backbone_gene_id | **Gene_id** | **Gene positions** | **Contig** | **Gene order** | **5'-3’ end** | **Gene distance** | **Domain score** | | | **Annotated gene function** |
| MKGM0000378_g24 | MKGM0000378_g25 | 1 | 378 | 5 | 607-15650 | 0 | 0 | | | Hydroxymate type ferrichrome siderophore peptide synthase |
| MKGM0000378_g24 | MKGM0000378_g24 | 0 | 378 | 4 | 17900-21654 | 1541 | 1 | | | L-ornithine N-5 monooxygenase |
| MKGM0000378_g24 | MKGM0000378_g23 | -1 | 378 | 3 | 24369-26700 | 1837 | 1 | | | Aspergillopepsin |
| MKGM0000378_g24 | MKGM0000378_g22 | -2 | 378 | 2 | 27351-28215 | 18923 | 1 | | | Mediator of RNA polymerase-II transcription subutit 7 |
| MKGM0000378_g24 | MKGM0000378_g21 | -3 | 378 | 1 | 30100-31838 | 961 | 1 | | | Hypothetical |
|  | | | | | | | | | | |
| *Ceratocystis platani* multimodular NRPS cluster - Contig 340 (LBBL00000340) | | | | | | | | | | |
| Backbone_gene_id | **Gene_id** | **Gene positions** | **Contig** | **Gene order** | **5'-3’ end** | **Gene distance** | **Domain score** | | | **Annotated gene function** |
| g17 | g18 | -1 | 340 | 7 | 21728-36752 | 131 | 1 | | | Hydroxymate type ferrichrome siderophore peptide synthase |
| g17 | g17 | 0 | 340 | 6 | 13709-15387 | 0 | 0 | | | L-ornithine N-5 monooxygenase |
| g17 | g14 | 3 | 340 | 3 | 11645-12922 | 3580 | 1 | | | Aspergillopepsin |
| g17 | g13 | 4 | 340 | 2 | 9105-9932 | 476 | 1 | | | Mediator of RNA polymerase-II transcription subutit 7 |
| g17 | g16 | 8 | 340 | 5 | 7508-8557 | 2250 | 0 | | | Hypothetical |
|  | | | | | | | | | | |
| *Ceratocystis smalleyi* multimodular NRPS cluster - Contig 288 (NETT01000228) | | | | | | | | | | |
| Backbone_gene_id | **Gene_id** | **Gene positions** | **Contig** | **Gene order** | **5'-3’ end** | **Gene distance** | **Domain score** | | | **Annotated gene function** |
| NETT01000228_g19 | NETT01000228_g20 | -1 | 228 | 11 | 24617-39671 | 0 | 1 | | | Hydroxymate type ferrichrome siderophore peptide synthase |
| NETT01000228_g19 | NETT01000228_g19 | 0 | 228 | 10 | 46319-47994 | 201 | 0 | | | L-ornithine N-5 monooxygenase |
| NETT01000228_g19 | NETT01000228_g17 | 2 | 228 | 8 | 48781-50058 | 429 | 0 | | | Aspergillopepsin |
| NETT01000228_g19 | NETT01000228_g16 | 3 | 228 | 7 | 51768-52595 | 303 | 0 | | | Mediator of RNA polymerase-II transcription subutit 7 |
| NETT01000228_g19 | NETT01000228_g18 | 3 | 228 | 9 | 53143-54198 | 815 | 1 | | | Hypothetical |
|  | | | | | | | | | | |
| *Huntiella decipiens* multimodular NRPS cluster - Contig 307 (NETU00000307) | | | | | | | | | | |
| Backbone_gene_id | **Gene_id** | **Gene positions** | **Contig** | **Gene order** | **5'-3’ end** | **Gene distance** | **Domain score** | | | **Annotated gene function** |
| NETU00000307_g13 | NETU00000307_g13 | 0 | 307 | 5 | 52009-66674 | 0 | 1 | | | Hydroxymate type ferrichrome siderophore peptide synthase |
| NETU00000307_g13 | NETU00000307_g11 | 2 | 307 | 3 | 42395-44050 | 2701 | 1 | | | L-ornithine N-5 monooxygenase |
| NETU00000307_g13 | NETU00000307_g10 | 3 | 307 | 2 | 40415-41656 | 914 | 1 | | | Endothiapepsin |
| NETU00000307_g13 | NETU00000307_g12 | 10 | 307 | 4 | 37016-37582 | 906 | 0 | | | Hypothetical |
| NETU00000307_g13 | NETU00000307_g10 | 5 | 307 | 1 | 31291-32148 | 1457 | 0 | | | Hypothetical |
| NETU00000307_g13 | NETU00000307_g12 | 7 | 307 | 8 | 30182-31007 | 1678 | 0 | | | Mediator of RNA polymerase-II transcription subutit 7 |
| NETU00000307_g13 | NETU00000307_g12 | 6 |  | 7 | 26531-29451 | 1956 | 1 | | | Membrane protein |
|  | | | | | | | | | | |
| *Huntiella bhutanensis* multimodular NRPS cluster - Contig 83 (MJMS00000083) | | | | | | | | | | |
| Backbone_gene_id | **Gene_id** | **Gene positions** | **Contig** | **Gene order** | **5'-3’ end** | **Gene distance** | **Domain score** | | | **Annotated gene function** |
| g27 | g24 | 3 | 83 | 1 | 111372-126028 | 24395 | 0 | | | Hydroxymate type ferrichrome siderophore peptide synthase |
| g27 | g25 | 2 | 83 | 2 | 101811-103465 | 17082 | 0 | | | L-ornithine N-5 monooxygenase |
| g27 | g26 | 1 | 83 | 3 | 99848-101089 | 5131 | 1 | | | Endothiapepsin |
| g27 | g27 | 0 | 83 | 4 | 96401-96964 | 0 | 0 | | | Hypothetical |
| g27 | g28 | -1 | 83 | 5 | 90635-91486 | 13958 | 0 | | | Hypothetical |
| g27 | g29 | -2 | 83 | 6 | 89531-90356 | 20223 | 0 | | | Mediator of RNA polymerase-II transcription subutit 7 |
|  | | | | | | | | | | |
| *Huntiella moniliformis* multimodular NRPS cluster - Contig 10 (JMSH00000010) | | | | | | | | | | |
| Backbone_gene_id | **Gene_id** | **Gene positions** | **Contig** | **Gene order** | **5'-3’ end** | **Gene distance** | **Domain score** | | | **Annotated gene function** |
| JMSH00000010_g19 | JMSH00000010_g17 | 3 | 10 | 2 | 384688-400320 | 481 | 0 | | | Hydroxymate type ferrichrome siderophore peptide synthase |
| JMSH00000010_g19 | JMSH00000010_g16 | 2 | 10 | 1 | 374979-376635 | 1739 | 1 | | | L-ornithine N-5 monooxygenase |
| JMSH00000010_g19 | JMSH00000010_g20 | 1 | 10 | 5 | 373044-374285 | 133 | 0 | | | Endothiapepsin |
| JMSH00000010_g19 | JMSH00000010_g19 | 0 | 10 | 4 | 369599-370160 | 0 | 0 | | | Hypothetical |
| JMSH00000010_g19 | JMSH00000010_g18 | -1 | 10 | 3 | 363872-364723 | 1373 | 0 | | | Hypothetical |
|  | | | | | | | | | | |
| *Huntiella omanensis* multimodular NRPS cluster - Contig 6485 (JSUI000006485) | | | | | | | | | | |
| Backbone_gene_id | **Gene_id** | **Gene positions** | **Contig** | **Gene order** | **5'-3’ end** | **Gene distance** | **Domain score** | | | **Annotated gene function** |
| JSUI000006485_g5 | JSUI000006485_g6 | -1 | 6485 | 7 | 1-15106 | 131 | 1 | | | Hydroxymate type ferrichrome siderophore peptide synthase |
| JSUI000006485_g5 | JSUI000006485_g5 | 0 | 6485 | 6 | 23511-25392 | 0 | 0 | | | L-ornithine N-5 monooxygenase |
| JSUI000006485_g5 | JSUI000006485_g2 | 3 | 6485 | 3 | 26138-27379 | 3580 | 1 | | | Endothiapepsin |
| JSUI000006485_g5 | JSUI000006485_g1 | 4 | 6485 | 2 | 31312-32710 | 476 | 1 | | | Hypothetical |
| JSUI000006485_g5 | JSUI000006485_g4 | 8 | 6485 | 5 | 38419-38937 | 2250 | 0 | | | Hypothetical |
| JSUI000006485_g5 | JSUI000006485_g3 | 9 | 6485 | 4 | 39947-41054 | 785 | 0 | | | Mediator of RNA polymerase-II transcription subutit 7 |
|  | | | | | | | | | | |
| *Huntiella savannae* multimodular NRPS cluster - Contig 52 (LCZG00000052) | | | | | | | | | | |
| Backbone_gene_id | **Gene_id** | **Gene positions** | **Contig** | **Gene order** | **5'-3’ end** | **Gene distance** | **Domain score** | | | **Annotated gene function** |
| LCZG00000052_g5 | LCZG00000052_g11 | -8 | 52 | 9 | 1-15106 | 0 | 1 | | | Hydroxymate type ferrichrome siderophore peptide synthase |
| LCZG00000052_g5 | LCZG00000052_g10 | -7 | 52 | 8 | 23511-25392 | 3089 | 1 | | | L-ornithine N-5 monooxygenase |
| LCZG00000052_g5 | LCZG00000052_g9 | -6 | 52 | 7 | 26138-27379 | 265 | 0 | | | Endothiapepsin |
| LCZG00000052_g5 | LCZG00000052_g8 | -5 | 52 | 6 | 31312-32710 | 27 | 0 | | | Hypothetical |
| LCZG00000052_g5 | LCZG00000052_g7 | -4 | 52 | 5 | 38419-38937 | 52 | 1 | | | Hypothetical |
| LCZG00000052_g5 | LCZG00000052_g6 | -3 | 52 | 4 | 39947-41054 | 421 | 0 | | | Mediator of RNA polymerase-II transcription subutit 7 |
| LCZG00000052_g5 | LCZG00000052_g5 | 0 | 52 | 3 | 42379-47068 | 684 | 0 | | | Membrane protein |
|  | | | | | | | | | | |
| *Thielaviopsis musarum* multimodular NRPS cluster - Contig 131 (LKBB00000131) | | | | | | | | | | |
| Backbone_gene_id | **Gene_id** | **Gene positions** | **Contig** | **Gene order** | **5'-3’ end** | **Gene distance** | **Domain score** | | | **Annotated gene function** |
| LKBB00000131_g44 | LKBB00000131_g44 | 0 | 131 | 4 | 4928-14284 | 0 | 1 | | | Hydroxymate type ferrichrome siderophore peptide synthase |
| LKBB00000131_g44 | LKBB00000131_g41 | 3 | 131 | 1 | 14427-14781 | 2611 | 1 | | | No similarity |
| LKBB00000131_g44 | LKBB00000131_g43 | 8 | 131 | 3 | 14895-19992 | 3258 | 1 | | | No similarity |
| LKBB00000131_g44 | LKBB00000131_g42 | 9 | 131 | 2 | 23912-25208 | 348 | 1 | | | L-ornithine N-5 monooxygenase |
| LKBB00000131_g44 | LKBB00000131_g4 | 5 | 131 | 5 | 25567-26826 | 1548 | 1 | | | Endothiapepsin |
|  | | | | | | | | | | |
| *Thielaviopsis punctulata* multimodular NRPS cluster - Contig 405 (LAEV00000405) | | | | | | | | | | |
| Backbone_gene_id | **Gene_id** | **Gene positions** | **Contig** | **Gene order** | **5'-3’ end** | **Gene distance** | **Domain score** | | | **Annotated gene function** |
| LAEV00000405_g3 | LAEV00000405_g5 | -3 | 405 | 6 | 44375-58733 | 0 | 1 | | | Hydroxymate type ferrichrome siderophore peptide synthase |
| LAEV00000405_g3 | LAEV00000405_g4 | -2 | 405 | 5 | 38397-40068 | 4450 | 0 | | | L-ornithine N-5 monooxygenase |
| LAEV00000405_g3 | LAEV00000405_g3 | -1 | 405 | 4 | 36850-38106 | 7008 | 0 | | | Endothiapepsin |
| LAEV00000405_g3 | LAEV00000405_g3 | 0 | 405 | 3 | 30987-32033 | 225 | 0 | | | Hypothetical |
| LAEV00000405_g3 | LAEV00000405_g1 | 1 | 405 | 2 | 29339-30675 | 24752 | 1 | | | F-box domain |
| LAEV00000405_g3 | LAEV00000405_g8 | 2 | 405 | 1 | 44375-58733 | 27509 | 0 | | | Hydroxymate type ferrichrome siderophore peptide synthase |
|  | | | | | | | | | | |
| *Endoconidiophora laricicola* multimodular NRPS cluster - Contig 376 (LXGT00000376) | | | | | | | | | | |
| Backbone_gene_id | **Gene_id** | **Gene positions** | **Contig** | **Gene order** | **5'-3’ end** | **Gene distance** | **Domain score** | | | **Annotated gene function** |
| LXGT00000376_g25 | LXGT00000376_g25 | 0 | 376 | 4 | 664-1427 | 3258 | 1 | | | Glutathione transferase |
| LXGT00000376_g25 | LXGT00000376_g26 | 3 | 376 | 1 | 6275-7575 | 2611 | 1 | | | Transposase |
| LXGT00000376_g25 | LXGT00000376_g27 | 8 | 376 | 3 | 8168-22785 | 0 | 1 | | | Hydroxymate type ferrichrome siderophore peptide synthase |
| LXGT00000376_g25 | LXGT00000376_g28 | 9 | 376 | 2 | 32170-33814 | 348 | 1 | | | L-ornithine N-5 monooxygenase |
| LXGT00000376_g25 | LXGT00000376_g23 | 7 | 376 | 6 | 35185-36432 | 568 | 0 | | | Endothiapepsin |
| LXGT00000376_g25 | LXGT00000376_g24 | 6 | 376 | 7 | 39445-40399 | 456 | 0 | | | Mediator of RNA polymerase-II transcription subutit 7 |
|  | | | | | | | | | | |
| *Endoconidiophora polonica* multimodular NRPS cluster - Contig 625 (LXKZ00000625) | | | | | | | | | | |
| Backbone_gene_id | **Gene_id** | **Gene positions** | **Contig** | **Gene order** | **5'-3’ end** | **Gene distance** | **Domain score** | | | **Annotated gene function** |
| g19 | g18 | -1 | 625 | 7 | 1-15495 | 131 | 1 | | | Hydroxymate type ferrichrome siderophore peptide synthase |
| g19 | g17 | 0 | 625 | 6 | 28287-29935 | 0 | 0 | | | L-ornithine N-5 monooxygenase |
| g19 | g14 | 3 | 625 | 3 | 30734-31996 | 3580 | 1 | | | Endothiapepsin |
| g19 | g13 | 4 | 625 | 2 | 36085--36931 | 476 | 1 | | | Mediator of RNA polymerase-II transcription subutit 7 |
| g19 | g16 | 8 | 625 | 5 | 37350-38432 | 2250 | 0 | | | Hypothetical |
|  | | | | | | | | | | |
| *Davidsoniella virescens* multimodular NRPS cluster - Contig 225 (LJZU000000225) | | | | | | | | | | |
| Backbone_gene_id | **Gene_id** | **Gene positions** | **Contig** | **Gene order** | **5'-3’ end** | **Gene distance** | **Domain score** | | | **Annotated gene function** |
| g24 | g24 | 3 | 225 | 1 | 68003-68824 | 24395 | 0 | | | F-box domain |
| g24 | g25 | 2 | 225 | 2 | 69398-70459 | 17082 | 0 | | | Hypothetical |
| g24 | g26 | 1 | 225 | 3 | 70871-71731 | 5131 | 1 | | | Transcription subunit 7 |
| g24 | g27 | 0 | 225 | 4 | 75705-76976 | 0 | 0 | | | Peptidase A1 |
| g24 | g28 | -1 | 225 | 5 | 77871-79530 | 13958 | 0 | | | L-ornithine N-5-monooxygenase |
| g24 | g29 | -2 | 225 | 6 | 90563-105723 | 20223 | 0 | | | NRPS |
|  | | | | | | | | | | |
| *Ambrosiella xylebori* multi modular NRPS Contig 4 (PCDO01000004) | | | | | | | | | | |
| Backbone_gene_id | **Gene_id** | **Gene positions** | **Contig** | **Gene order** | **5'-3’ end** | **Gene distance** | **Domain score** | | | **Annotated gene function** |
| PCDO01000004_g12 | PCDO01000004_g13 | 0 | 4 | 5 | 27849-31063 | 0 | 1 | | | HIR 1 |
| PCDO01000004_g12 | PCDO01000004_g11 | 2 | 4 | 3 | 33739-35426 | 2701 | 1 | | | UDP-galactose transporter |
| PCDO01000004_g12 | PCDO01000004_g10 | 3 | 4 | 2 | 35787-37176 | 914 | 1 | | | Mannose-1-phosphate guanylyltransferase |
| PCDO01000004_g12 | PCDO01000004_g12 | 10 | 4 | 4 | 49879-64319 | 906 | 0 | | | NRPS |
| PCDO01000004_g12 | PCDO01000004_g10 | 5 | 4 | 1 | 73111-74807 | 1457 | 0 | | | L-ornithine 5-monooxygenase |
|  | | | | | | | | | | |
| *Davidsoniella australis* multi modular NRPS contig 143 | | | | | | | | | | |
| Backbone_gene_id | **Gene_id** | **Gene positions** | **Contig** | **Gene order** | **5'-3’ end** | **Gene distance** | **Domain score** | | | **Annotated gene function** |
| g15 | g21 | 1 | 143 | 6 | 6309-7485 | 473 | 0 | | | Ergosterol biosynthetic protein 28 |
| g15 | g20 | 2 | 143 | 5 | 7946-8525 | 699 | 1 | | | Galactose-1-phosphate uridylyltransferase |
| g15 | g18 | 3 | 143 | 4 | 8616-10034 | 3613 | 0 | | | Transcriptional adapter 2 |
| g15 | g17 | 4 | 143 | 3 | 10992-12653 | 130 | 1 | | | Hypothetical |
| g15 | g16 | 5 | 143 | 2 | 14724-15219 | 606 | 0 | | | HD Domain |
| g15 | g15 | 6 | 143 | 1 | 18529-33759 | 0 | 1 | | | NRPS |
|  | | | | | | | | | | |
| *Berkeleyomyces basicola* multimodular NRPS cluster - Contig 12 | | | | | | | | | | |
| Backbone_gene_id | **Gene_id** | **Gene positions** | **Contig** | **Gene order** | **5'-3’ end** | **Gene distance** | **Domain score** | | | **Annotated gene function** |
| g24 | g25 | 1 | 12 | 5 | 1-1493 | 0 | 0 | | | Hypothetical |
| g24 | g24 | 0 | 12 | 4 | 6645-21513 | 1541 | 1 | | | NRPS |
| g24 | g23 | -1 | 12 | 3 | 27720-29384 | 1837 | 1 | | | L-ornithine 5-monooxygenase |
| g24 | g22 | -2 | 12 | 2 | 31358-32199 | 18923 | 1 | | | RNA polymerase II subunit 7 |
| g24 | g21 | -3 | 12 | 1 | 43568-44221 | 961 | 1 | | | Hypothetical |
