# Supplementary material for: Distribution and Evolution of Nonribosomal Peptide Synthetase Gene Clusters in the Ceratocystidaceae
Source: Genes (Basel). 2019 Apr 30;10(5):328. doi: 10.3390/genes10050328 (PMC6563098; doi:10.3390/genes10050328)
Supplement: Supplementary file 1 [file genes-10-00328-s001.zip › Supplementary Files/Supplementary file S3 Feb 2019.docx]

### **SUPPLEMENTARY FILE S3.**

*(Sayari et al - Ceratocystidaceae Nonribosomal peptide synthetase gene clusters)*

### Blast hits for putative *Ceratocystidaceae* NRPS biosynthetic gene clusters predicted with antiSMASH. For confirmation of the antiSMASH results, we utilized SMURF (www.jcvi.org/smurf/; (Khaldi et al. 2010). For this purpose, genes that were 15Kb upstream and downstream of the identified NRPS genes, were retrieved and submitted to the BLASTp server at National Centre for Biotechnology Information (NCBI, ftp://ftp.ncbi.nih.gov/blast/) for identification.

| A-1) *Ceratocystis adiposa* monomodular NRPS cluster_Contig127 (LXGU00000127) | | | | |  |  |  |
| --- | --- | --- | --- | --- | --- | --- | --- |
| Gene name | Size (aa) | Location on the contig | Species | E value | % Coverage | % identity | Accession number of top blast hit |
| Ras_GTPase | 208 | 10101-11196 | *Grosmannia clavigera* | 9e-142 | 96 | 98 | XP_014176081 |
| Peroxin 14/17 | 374 | 12451-13304 | Drechmeria*coniospora* | 1e-42 | 99 | 34 | KYK54231 |
| Hypothetical | 799 | 17267-18477 | *Metarhizium rileyi* | 4e-45 | 40 | 41 | OAA44328 |
| Hypothetical | 562 | 19710-23430 | *Stachybotrys chartarum* | 8e-31 | 50 | 31 | KFA73722 |
| NRPS | 2092 | 29439-31379 | *Colletotrichum gloeosporioides* | 0 | 87 | 55 | XP_007284335 |
| CoA transferase | 577 | 32137-38636 | *Neonectria ditissima* | 0 | 98 | 75 | KPM45703 |
| Siderophore transporter | 586 | 44922-46799 | *Trichoderma quizhouense* | 0 | 96 | 55 | OPB43222 |
| Siderophore biosynthesis | 452 | 48845-50814 | *Purpureocillium lilacinum* | 0 | 99 | 64 | OAQ83990 |
| Oxidoreductase | 421 | 52470-53828 | *Trichoderma quizhouense* | 5e-156 | 98 | 53 | OPB43223 |
| ABC-multidrug trasporter | 1370 | 53943-55716 | *Purpureocillium lilacinum* | 0 | 97 | 57 | OAQ83993 |
| Transporter | 832 | 65836-68393 | *Colletotrichum incanum* | 0 | 100 | 57 | OHW98426 |
| Geranylgeranyl transferase | 322 | 70784-71997 | *Colletotrichum salicis* | 1e-80 | 97 | 43 | KXH54021 |

| A-2) *Ceratocystis adiposa* multi-modular NRPS cluster_Contig 175 (LXGU00000175) | | | | |  |  |  |
| --- | --- | --- | --- | --- | --- | --- | --- |
| Gene name | Size (aa) | Location on the contig | Species | E value | % Coverage | % identity | Accession number of top blast hit |
| Glutathione transferase | 232 | 664-1427 | *Umbilicaria pustulata* | 3e-26 | 86 | 37 | SLM39268 |
| Transposase | 333 | 6275-7575 | *Fusarium oxysporum* | 7e-133 | 88 | 70 | ENH66637 |
| Hydroxymate type ferrichrome siderophore peptide synthase | 4810 | 8168-22785 | *Scedosporium apiospermum* | 0 | 99 | 40 | XP_016639834 |
| L-ornithine N-5 monooxygenase | 523 | 32170-33814 | *Fusarium avenaceum* | 0 | 91 | 59 | KIL93640 |
| Endothiapepsin | 415 | 35185-36432 | *Nectria ditissima* | 0 | 100 | 62 | KPM38239 |
| Mediator of RNA polymerase-II transcription subutit 7 | 297 | 39445-40399 | *Fusarium poae* | 3e-77 | 83 | 51 | OBS21164 |
| Hypothetical | 323 | 40811-41782 | *Fusarium langsethiae* | 9e-28 | 82 | 41 | KPA42345 |

| B-1) *Ceratocystis albifundus* monomodular NRPS cluster_Contig 1154 (JSSU000001154) | | | | |  |  |  |
| --- | --- | --- | --- | --- | --- | --- | --- |
| Gene name | Size | Location on the contig | Species | E value | % Coverage | % identity | Accession number of top blast hit |
| Glutamine synthase | 684 | 470-3248 | *Metarhizium brunneum* | 0 | 99 | 69 | XP_014545497 |
| Geranylgeranyl transferase | 316 | 3503-4522 | *Tolypocladium ophioglossoides* | 2e-97 | 98 | 47 | KND94315 |
| Transporter | 795 | 5694-8351 | *Colletotrichum tofieldiae* | 0 | 98 | 72 | KZL78304 |
| ABC-transporter | 1363 | 9455-13612 | *Colletotrichum chlorophyti* | 0 | 97 | 57 | OLN95357 |
| Oxidoreductase | 420 | 15070-16498 | *Trichoderma atroviride* | 1e-162 | 96 | 55 | XP_013943858 |
| Siderophore biosynthesis | 450 | 16684-18104 | *Drechmeria coniospora* | 0 | 99 | 62 | ODA83897 |
| Siderophore transporter | 1151 | 18824-23219 | *Trichoderma reesei* | 0 | 47 | 75 | XP_006968099 |
| NRPS | 2100 | 25279-31641 | *Colletotrichum gloeosporioides* | 0 | 88 | 53 | EQB59356 |
| Hypothetical | 595 | 33229-35016 | *Colletotrichum graminicola* | 2e-82 | 75 | 40 | XP_008096956 |

| B-2) *Ceratocystis albifundus* multi-modular NRPS cluster-Contig1169 (JSSU000001169) | | | | |  |  |  |
| --- | --- | --- | --- | --- | --- | --- | --- |
| Gene name | Size | Location on the contig | Species | E value | % Coverage | % identity | Accession number of top blast hit |
| Hydroxymate type ferrichrome siderophore peptide synthase | 4919 | 2141-17203 | *Scedosporium apiospermum* | 0 | 99 | 42 | XP_016639834 |
| L-ornithine N-5 monooxygenase | 530 | 23649-25327 | *Fusarium graminearum* | 0 | 99 | 55 | XP_011323895 |
| Mediator of RNA polymerase-II transcription subutit 7 | 255 | 28185-29012 | *Nectria haematococca* | 1e-87 | 98 | 54 | XP_003044025 |
| Hypothetical | 348 | 29567-30613 | *Colletotrichum salicis* | 3e-59 | 84 | 45 | KXH53360 |

| C-1) *Ceratocystis eucalypticola* monomodular NRPS cluster-Contig167 (LJOA00000167) | | | | |  |  |  |
| --- | --- | --- | --- | --- | --- | --- | --- |
| Gene name | Size | Location on the contig | Species | E value | % Coverage | % identity | Accession number of top blast hit |
| MFS-type transporter | 621 | 114070-116062 | *Neonectria ditissima* | 0 | 93 | 69 | KPM45342 |
| Glutamine synthase | 1172 | 119620-124574 | *Metarhizium brunneum* | 0 | 99 | 69 | XP_014545497 |
| ABC multidrug transporter | 2231 | 125712-133609 | *Pochonia chlamydospora* | 0 | 98 | 60 | XP_018147652 |
| Oxidoreductase | 420 | 135058-136469 | *Scedosporium apiospermum* | 8e-155 | 98 | 56 | XP_016644515 |
| Siderophore biosynthesis | 450 | 136660-138080 | *Colletotrichum orchidophilum* | 0 | 98 | 60 | XP_022473331 |
| Siderophore transporter | 1155 | 138773-143113 | *Trichoderma reesei* | 0 | 48 | 75 | XP_006968099 |
| NRPS | 2100 | 145186-151548 | *Colletotrichum gloeosporioides* | 0 | 88 | 53 | EQB59356 |
| Hypothetical | 597 | 153090-154483 | *Pochonia chlamydosporia* | 1e-81 | 75 | 40 | XP_018147657 |

| C-2) *Ceratocystis eucalypticola* multi-modular NRPS cluster-Contig 206 (LJOA00000206) | | | | |  |  |  |
| --- | --- | --- | --- | --- | --- | --- | --- |
| Gene name | Size | Location on the contig | Species | E value | % Coverage | % identity | Accession number of top blast hit |
| Hypothetical | 351 | 7544-8599 | *Colletotrichum salicis* | 4e-61 | 85 | 45 | KXH53360 |
| Mediator of RNA polymerase-II transcription subutit 7 | 255 | 9147-9974 | *Nectria haematococca* | 2e-85 | 98 | 53 | XP_003044025 |
| Aspergillopepsin | 425 | 11685-12962 | *Ceratocystis fimbriata* | 0 | 100 | 97 | PHH49949 |
| L-ornithine N-5 monooxygenase | 530 |  | *Fusarium graminearum* | 0 | 99 | 55 | XP_011323895 |
| Hydroxymate type ferrichrome siderophore peptide synthase | 4918 | 21836-36887 | *Scedosporium apiospermum* | 0 | 99 | 42 | XP_016639834 |

| D-1) *Bretziella fagacearum* monomodular NRPS cluster_Contig 3 (MKGJ00000003) | | | | |  |  |  |
| --- | --- | --- | --- | --- | --- | --- | --- |
| Gene name | Size | Location on the contig | Species | E value | % Coverage | % identity | Accession number of top blast hit |
| Geranylgeranyl transferase | 330 | 13506-14685 | *Magnaporthe poae* | 7e-110 | 79 | 61 | KLU91995 |
| Transporter | 932 | 16580-19884 | *Colletotrichum gloeosporioides* | 0 | 96 | 69 | XP_007273195 |
| ABC-transporter | 1374 | 20758-24989 | *Metarhizium acridum* | 0 | 99 | 55 | XP_007808197 |
| Oxidoreductase | 420 | 28003-29707 | *Metarhizium rileyi* | 4e-145 | 96 | 56 | OAA44322 |
| Siderophore biosynthesis | 452 | 29922-31280 | *Purpureocillium lilacinum* | 0 | 99 | 65 | OAQ83990 |
| Siderophore transporter | 555 | 33090-34757 | *Trichoderma quizhouense* | 0 | 100 | 56 | OPB43222 |
| Acyl-CoA synthetase | 577 | 36234-38121 | *Nectria ditissima* | 0 | 98 | 75 | KPM45703 |
| NRPS | 1960 | 41315-47342 | *Colletotrichum gloeosporioides* | 0 | 96 | 54 | EQB59356 |
| Hypothetical | 595 | 47969-50689 | *Thielavia terrestris* | 2e-65 | 67 | 37 | XP_003657661 |

| D-2) *Bretziella fagacearum* multi-modular NRPS cluster-Contig 227 (MKGJ00000227) | | | | |  |  |  |
| --- | --- | --- | --- | --- | --- | --- | --- |
| Gene name | Size | Location on the contig | Species | E value | % Coverage | % identity | Accession number of top blast hit |
| Hydroxymate type ferrichrome siderophore peptide synthase | 4766 | 1-14960 | *Scedosporium apiospermum* | 0 | 99 | 42 | XP_016639834 |
| L-ornithine N-5 monooxygenase | 527 | 21912-23581 | *Purpureocillium lilacinum* | 0 | 89 | 60 | XP_018181246 |
| 1,4 glucanase | 41 | 24912-26018 | *Neonectria ditissima* | 2e-130 | 99 | 55 | KPM42114 |
| Chitinase | 714 | 28750-31021 | *Valsa mali* | 6e-23 | 42 | 43 | KUI56008 |
| Hypothetical | 618 | 35659-37651 | *Thielavia terrestris* | 2e-65 | 67 | 37 | XP_003657661 |

| E-1) *Ceratocystis manginecans* monomodular NRPS cluster-Contig 3 ([JJRZ00000000](http://www.ncbi.nlm.nih.gov/nuccore/659897781)3) | | | | |  |  |  |
| --- | --- | --- | --- | --- | --- | --- | --- |
| Gene name | Size | Location on the contig | Species | E value | % Coverage | % identity | Accession number of top blast hit |
| Hypothetical | 597 | 1888-3681 | *Pochonia chlamydosporia* | 1e-81 | 75 | 40 | XP_018147657 |
| NRPS | 2100 | 5223-11585 | *Colletotrichum gloeosporioides* | 0 | 86 | 53 | XP_007284335 |
| Siderophore transporter | 1158 | 13658-17997 | *Trichoderma reesei* | 0 | 48 | 75 | XP_006968099 |
| Siderophore biosynthesis | 450 | 18684-20104 | *Colletotrichum orchidophilum* | 0 | 98 | 60 | XP_022473331 |
| Oxidoreductase | 420 | 20295-21706 | *Scedosporium apiospermum* | 5e-155 | 97 | 56 | XP_016644515 |
| ABC-multidrug trasporter | 2231 | 23155-31053 | *Pochonia chlamydosporia* | 0 | 98 | 60 | XP_018147652 |
| Glutamine synthase | 1147 | 32191-37148 | *Metarhizium brunneum* | 0 | 61 | 71 | XP_014545497 |

| E-2) *Ceratocystis manginecans* multi-modular NRPS cluster-Contig 189 ([JJRZ000000189](http://www.ncbi.nlm.nih.gov/nuccore/659897781)) | | | | |  |  |  |
| --- | --- | --- | --- | --- | --- | --- | --- |
| Gene name | Size | Location on the contig | Species | E value | % Coverage | % identity | Accession number of top blast hit |
| Hydroxymate type ferrichrome siderophore peptide synthase | 4919 | 24617-39671 | *Scedosporium apiospermum* | 0 | 99 | 42 | XP_016639834 |
| L-ornithine N-5 monooxygenase | 530 | 46319-47994 | *Fusarium graminearum* | 0 | 99 | 55 | XP_011323895 |
| Aspergillopepsin | 425 | 48781-50058 | *Ceratocystis fimbriata* | 0 | 100 | 97 | PHH49949 |
| Mediator of RNA polymerase-II transcription subutit 7 | 255 | 51768-52595 | *Nectria haematococca* | 4e-86 | 98 | 54 | XP_003044025 |
| Hypothetical | 351 | 53143-54198 | *Colletotrichum salicis* | 4e-61 | 85 | 45 | KXH53360 |

| F-1) *Ceratocystis fimbriata* monomodular NRPS cluster-Contig 521 ([APWK00000521)](http://www.ncbi.nlm.nih.gov/nuccore/APWK00000000) | | | | |  |  |  |
| --- | --- | --- | --- | --- | --- | --- | --- |
| Gene name | Size (aa) | Location on the contig | Species | E value | % Coverage | % identity | Accession number of top blast hit |
| NRPS | 2100 | 848-7210 | *Colletotrichum gloeosporioides* | 0 | 88 | 53 | XP_007284335 |
| Hypothetical | 597 | 8963-10756 | *Pochonia chlamydosporia* | 1e-81 | 75 | 40 | XP_018147657 |
| Siderophore transporter | 1158 | 16157-17422 | *Trichoderma reesei* | 0 | 48 | 75 | XP_006968099 |
| Siderophore biosynthesis | 451 | 22831-24020 | *Colletotrichum orchidophilum* | 0 | 97 | 59 | XP_022473331 |
| Oxidoreductase | 420 | 25221-26372 | *Scedosporium apiospermum* | 5e-1152 | 96 | 55 | XP_016644515 |
| ABC-multidrug trasporter | 2230 | 27634-30122 | *Pochonia chlamydosporia* | 0 | 98 | 60 | XP_018147652 |
| Glutamine synthase | 1145 | 31054-32961 | *Metarhizium brunneum* | 0 | 60 | 70 | XP_014545497 |

| F-2) *Ceratocystis fimbriata* multi-modular NRPS cluster-Contig 182 ([APWK00000182)](http://www.ncbi.nlm.nih.gov/nuccore/APWK00000000) | | | | |  |  |  |
| --- | --- | --- | --- | --- | --- | --- | --- |
| Gene name | Size (aa) | Location on the contig | Species | E value | % Coverage | % identity | Accession number of top blast hit |
| Hydroxymate type ferrichrome siderophore peptide synthase | 4919 | 24617-39671 | *Scedosporium apiospermum* | 0 | 99 | 42 | XP_016639834 |
| L-ornithine N-5 monooxygenase | 530 | 46319-47994 | *Fusarium graminearum* | 0 | 99 | 55 | XP_011323895 |
| Aspergillopepsin | 425 | 48781-50058 | *Ceratocystis fimbriata* | 0 | 100 | 97 | PHH49949 |
| Mediator of RNA polymerase-II transcription subutit 7 | 255 | 51768-52595 | *Nectria haematococca* | 4e-86 | 98 | 54 | XP_003044025 |
| Hypothetical | 351 | 53143-54198 | *Colletotrichum salicis* | 4e-61 | 85 | 45 | KXH53360 |

| G-1) *Ceratocystis harringtonii* monomodular NRPS cluster-Contig 60 (MKGM0000060) | | | | |  |  |  |
| --- | --- | --- | --- | --- | --- | --- | --- |
| Gene name | Size | Location on the contig | Species | E value | % Coverage | % identity | Accession number of top blast hit |
| Hypothetical | 597 | 75458-77251 | *Pochonia chlamydosporia* | 4e-82 | 75 | 40 | XP_018147657 |
| NRPS | 2100 | 78788-85150 | *Colletotrichum gloeosporioides* | 0 | 87 | 53 | XP_007284335 |
| Siderophore transporter | 1116 | 87218-93614 | *Trichoderma reesei* | 0 | 35 | 74 | XP_006968099 |
| Siderophore biosynthesis | 450 | 93808-95231 | *Colletotrichum orchidophilum* | 0 | 98 | 60 | XP_022473331 |
| Oxidoreductase | 429 | 96635-98124 | *Scedosporium apiospermum* | 1e-164 | 97 | 56 | XP_016644515 |
| ABC-multidrug trasporter | 2231 | 100363-107756 | *Pochonia chlamydosporia* | 0 | 98 | 60 | XP_018147652 |
| Glutamine synthase | 1147 | 108239-110589 | *Metarhizium brunneum* | 0 | 61 | 71 | XP_014545497 |

| G-2) *Ceratocystis harringtonii* multi-modular NRPS cluster-Contig 378 (MKGM0000378) | | | | |  |  |  |
| --- | --- | --- | --- | --- | --- | --- | --- |
| Gene name | Size | Location on the contig | Species | E value | % Coverage | % identity | Accession number of top blast hit |
| Hydroxymate type ferrichrome siderophore peptide synthase | 4919 | 607-15650 | *Scedosporium apiospermum* | 0 | 99 | 42 | XP_016639834 |
| L-ornithine N-5 monooxygenase | 530 | 17900-21654 | *Fusarium graminearum* | 0 | 99 | 55 | XP_011323895 |
| Aspergillopepsin | 425 | 24369-26700 | *Ceratocystis fimbriata* | 0 | 100 | 97 | PHH49949 |
| Mediator of RNA polymerase-II transcription subutit 7 | 255 | 27351-28215 | *Nectria haematococca* | 4e-86 | 98 | 54 | XP_003044025 |
| Hypothetical | 351 | 30100-31838 | *Colletotrichum salicis* | 4e-61 | 85 | 45 | KXH53360 |

| H-1) *Ceratocystis platani* monomodular NRPS cluster-Contig 109 (LBBL00000109) | | | | |  |  |  |
| --- | --- | --- | --- | --- | --- | --- | --- |
| Gene name | Size | Location on the contig | Species | E value | % Coverage | % identity | Accession number of top blast hit |
| Transposable element | 403 | 1-1320 | *Fusarium oxysporum* | 2e-129 | 93 | 68 | ENH65995 |
| Hypothetical | 204 | 7955-8569 | *Pochonia chlamydosporia* | 1e-81 | 75 | 40 | XP_018147657 |
| Glutamine synthase | 1370 | 14397-19936 | *Metarhizium brunneum* | 0 | 51 | 71 | XP_014545497 |
| ABC-multidrug trasporter | 2233 | 21080-28988 | *Pochonia chlamydosporia* | 0 | 98 | 60 | XP_018147652 |
| Oxidoreductase | 420 | 30437-31848 | *Scedosporium apiospermum* | 4e-155 | 97 | 56 | XP_016644515 |
| Siderophore biosynthesis | 450 | 32039-38464 | *Colletotrichum orchidophilum* | 0 | 98 | 60 | XP_022473331 |
| Siderophore transporter | 1618 | 40639-47001 | *Trichoderma reesei* | 0 | 34 | 76 | XP_006968099 |
| NRPS | 2100 | 48540-50333 | *Colletotrichum gloeosporioides* | 0 | 88 | 53 | XP_007284335 |
| Hypothetical | 597 | 53928-55020 | *Pochonia chlamydosporia* | 1e-81 | 75 | 40 | XP_018147657 |
| Hypothetical | 306 | 55777-57041 | *Stachybotrys chlorohalonata* | 6e-48 | 99 | 48 | KFA61745 |

| H-2) *Ceratocystis platani* multi-modular NRPS cluster-Contig 340 (LBBL00000340) | | | | |  |  |  |
| --- | --- | --- | --- | --- | --- | --- | --- |
| Gene name | Size | Location on the contig | Species | E value | % Coverage | % identity | Accession number of top blast hit |
| Hydroxymate type ferrichrome siderophore peptide synthase | 4909 | 21728-36752 | *Scedosporium apiospermum* | 0 | 99 | 42 | XP_016639834 |
| L-ornithine N-5 monooxygenase | 530 | 13709-15387 | *Fusarium graminearum* | 0 | 99 | 55 | XP_011323895 |
| Aspergillopepsin | 425 | 11645-12922 | *Ceratocystis fimbriata* | 0 | 100 | 96 | PHH49949 |
| Mediator of RNA polymerase-II transcription subutit 7 | 255 | 9105-9932 | *Nectria haematococca* | 2e-87 | 98 | 54 | XP_003044025 |
| Hypothetical | 349 | 7508-8557 | *Colletotrichum salicis* | 1e-60 | 85 | 45 | KXH53360 |

| I-1) *Ceratocystis smalleyi* monomodular NRPS cluster-Contig 330 (NETT01000330) | | | | |  |  |  |
| --- | --- | --- | --- | --- | --- | --- | --- |
| Gene name | Size | Location on the contig | Species | E value | % Coverage | % identity | Accession number of top blast hit |
| Hypothetical | 349 | 20919-22712 | *Pochonia chlamydosporia* | 9e-81 | 75 | 40 | XP_018147657 |
| NRPS | 2100 | 13000-19362 | *Colletotrichum gloeosporioides* | 0 | 87 | 53 | XP_007284335 |
| Siderophore transporter | 1155 | 6583-10915 | *Trichoderma reesei* | 0 | 48 | 75 | XP_006968099 |
| Siderophore biosynthesis | 85 | 6200-6485 | *Colletotrichum orchidophilum* | 0 | 98 | 60 | XP_022473331 |
| Oxidoreductase | 420 | 5100-6123 | *Scedosporium apiospermum* | 5e-155 | 97 | 56 | XP_016644515 |
| ABC-multidrug trasporter | 1231 | 1-4863 | *Pochonia chlamydosporia* | 0 | 98 | 60 | XP_018147652 |

| I-2) *Ceratocystis smalleyi* multi-modular NRPS cluster-Contig 288 (NETT01000228) | | | | |  |  |  |
| --- | --- | --- | --- | --- | --- | --- | --- |
| Gene name | Size | Location on the contig | Species | E value | % Coverage | % identity | Accession number of top blast hit |
| Hydroxymate type ferrichrome siderophore peptide synthase | 4920 | 24617-39671 | *Scedosporium apiospermum* | 0 | 99 | 42 | XP_016639834 |
| L-ornithine N-5 monooxygenase | 530 | 46319-47994 | *Fusarium graminearum* | 0 | 88 | 60 | XP_011323895 |
| Aspergillopepsin | 164 | 48781-50058 | *Ceratocystis fimbriata* | 2e-57 | 100 | 97 | PHH49949 |
| Mediator of RNA polymerase-II transcription subutit 7 | 255 | 51768-52595 | *Nectria haematococca* | 5e-87 | 98 | 54 | XP_003044025 |
| Hypothetical | 354 | 53143-54198 | *Colletotrichum salicis* | 2e-58 | 83 | 45 | KXH53360 |

| J-1) *Huntiella decipiens* monomodular NRPS cluster_Contig 65 (NETU00000065) | | | | |  |  |  |
| --- | --- | --- | --- | --- | --- | --- | --- |
| Gene name | Size (aa) | Location on the contig | Species | E value | % Coverage | % identity | Accession number of top blast hit |
| Hypothetical | 662 | 153578-156583 | *Colletotrichum nymphaeae* | 8e-62 | 65 | 35 | KXH42342 |
| NRPS | 2077 | 156800-163089 | *Colletotrichum gloeosporioides* | 0 | 90 | 54 | XP_007284335 |
| CoA transferase | 577 | 166514-168357 | *Neotectria ditissima* | 0 | 98 | 74 | KPM45703 |
| Siderophore transporter | 585 | 169239-170996 | *Trichoderma quizhouense* | 0 | 94 | 54 | OPB43222 |
| Siderophore biosynthesis | 451 | 171835-173190 | *Purpureocillium lilacinum* | 0 | 99 | 62 | OAQ83990 |
| Oxidoreductase | 430 | 173298-174886 | *Trichoderma quizhouense* | 3e-144 | 97 | 51 | OPB43223 |
| ABC-multidrug trasporter | 1363 | 178036-182188 | *Purpureocillium lilacinum* | 0 | 96 | 58 | OAQ83993 |
| Transporter | 971 | 182594-185767 | *Colletotrichum gloeosporioides* | 0 | 94 | 67 | XP_007273195 |
| Geranylgeranyl transferase | 322 | 186098-187816 | *Colletotrichum salicis* | 1e-80 | 97 | 43 | KXH54021 |

| J-2) *Huntiella decipiens* multi-modular NRPS cluster_Contig 307 (NETU00000307) | | | | |  |  |  |
| --- | --- | --- | --- | --- | --- | --- | --- |
| Gene name | Size (aa) | Location on the contig | Species | E value | % Coverage | % identity | Accession number of top blast hit |
| Hydroxymate type ferrichrome siderophore peptide synthase | 4782 | 52009-66674 | *Scedosporium apiospermum* | 0 | 99 | 40 | XP_016639834 |
| L-ornithine N-5 monooxygenase | 528 | 42395-44050 | *Fusarium oxysporum* | 0 | 99 | 55 | PCD43854 |
| Endothiapepsin | 413 | 40415-41656 | *Nectria ditissima* | 0 | 100 | 58 | KPM38239 |
| Hypothetical | 153 | 37016-37582 | *Scedosporium apiospermum* |  | 89 | 36 | XP_016639846 |
| Hypothetical | 285 | 31291-32148 | *Fusarium avenaceum* |  | 96 | 35 | KIL93607 |
| Mediator of RNA polymerase-II transcription subutit 7 | 297 | 30182-31007 | *Nectria haematococca* | 4e-85 | 95 | 45 | XP_003044025 |
| Membrane protein | 323 | 26531-29451 | *Colletotrichum graminicola* | 0 | 89 | 53 | XP_008095397 |

| K-1) *Huntiella bhutanensis* monomodular NRPS cluster_Contig 96 (MJMS00000096) | | | | |  |  |  |
| --- | --- | --- | --- | --- | --- | --- | --- |
| Gene name | Size (aa) | Location on the contig | Species | E value | % Coverage | % identity | Accession number of top blast hit |
| Hypothetical | 649 | 28120-30368 | *Verticillium longisporum* | 7e-64 | 67 | 40 | CRK10802 |
| NRPS | 2081 | 30585-36886 | *Colletotrichum gloeosporioides* | 0 | 90 | 54 | XP_007284335 |
| CoA transferase | 577 | 40294-42137 | *Neotectria ditissima* | 0 | 98 | 74 | KPM45703 |
| Siderophore transporter | 585 | 43020-44777 | *Trichoderma quizhouense* | 0 | 94 | 53 | OPB43222 |
| Siderophore biosynthesis | 451 | 45614-46969 | *Purpureocillium lilacinum* | 0 | 99 | 62 | OAQ83990 |
| Oxidoreductase | 430 | 47077-48665 | *Trichoderma quizhouense* | 9e-149 | 97 | 51 | OPB43223 |
| ABC-multidrug trasporter | 1363 | 51817-55969 | *Purpureocillium lilacinum* | 0 | 98 | 57 | OAQ83993 |
| Transporter | 1379 | 56375-61590 | *Scedosporium apiospermum* | 0 | 93 | 56 | XP_016644513 |
| Oxidoreductase | 432 | 61637-62985 | *Colletotrichum gloeosporioides* | 7e-98 | 96 | 42 | KXH54021 |
| N-Acyltransferase | 257 | 63280-64165 | *Aschersonia aleyrodis* | 8e-62 | 86 | 44 | KZZ98783 |
| Glutamine synthase | 722 | 65142-67536 | *Colletotrichum salicis* | 0 | 98 | 72 | KXH63435 |

| K-2) *Huntiella bhutanensis* multi-modular NRPS cluster_Contig 83 (MJMS00000083) | | | | |  |  |  |
| --- | --- | --- | --- | --- | --- | --- | --- |
| Gene name | Size (aa) | Location on the contig | Species | E value | % Coverage | % identity | Accession number of top blast hit |
| Hydroxymate type ferrichrome siderophore peptide synthase | 4782 | 111372-126028 | *Scedosporium apiospermum* | 0 | 99 | 40 | XP_016639834 |
| L-ornithine N-5 monooxygenase | 528 | 101811-103465 | *Fusarium oxysporum* | 0 | 99 | 55 | PCD43854 |
| Endothiapepsin | 413 | 99848-101089 | *Nectria ditissima* | 2e-167 | 100 | 58 | KPM38239 |
| Hypothetical | 152 | 96401-96964 | *Scedosporium apiospermum* | 8e-17 | 77 | 39 | XP_016639846 |
| Hypothetical | 283 | 90635-91486 | *Fusarium avenaceum* |  | 96 | 35 | KIL93607 |
| Mediator of RNA polymerase-II transcription subutit 7 | 256 | 89531-90356 | *Nectria haematococca* | 1e-82 | 95 | 52 | XP_003044025 |
| Membrane protein | 954 | 85883-88797 | *Colletotrichum tofieldiae* | 0 | 89 | 52 | KZL73724 |
| Hydrolase | 227 | 84610-85545 | *Trichoderma harzianum* | 0 | 97 | 60 | KKP04280 |

| L-1) *Huntiella moniliformis* monomodular NRPS cluster_Contig 76 (JMSH00000076) | | | | |  |  |  |
| --- | --- | --- | --- | --- | --- | --- | --- |
| Gene name | Size (aa) | Location on the contig | Species | E value | % Coverage | % identity | Accession number of top blast hit |
| Hypothetical | 649 | 65356-67601 | *Verticillium longisporum* | 2e-60 | 67 | 39 | CRK10802 |
| NRPS | 2081 | 67818-74119 | *Colletotrichum gloeosporioides* | 0 | 90 | 54 | XP_007284335 |
| CoA transferase | 577 | 77591-79435 | *Neotectria ditissima* | 0 | 98 | 74 | KPM45703 |
| Siderophore transporter | 585 | 80316-82073 | *Trichoderma quizhouense* | 0 | 94 | 52 | OPB43222 |
| Siderophore biosynthesis | 451 | 82913-84268 | *Purpureocillium lilacinum* | 0 | 99 | 62 | OAQ83990 |
| Oxidoreductase | 421 | 84374-85964 | *Trichoderma quizhouense* | 5e-148 | 97 | 52 | OPB43223 |
| ABC-multidrug trasporter | 1363 | 89140-93292 | *Purpureocillium lilacinum* | 0 | 98 | 57 | OAQ83993 |
| Transporter | 1243 | 94260-98918 | *Scedosporium apiospermum* | 0 | 99 | 58 | XP_016644513 |
| Oxidoreductase | 432 | 98965-101492 | *Colletotrichum gloeosporioides* | 1e-100 | 96 | 43 | KXH54021 |
| Glutamine synthase | 722 | 102448-104841 | *Colletotrichum salicis* | 0 | 98 | 72 | KXH63435 |

| L-2) *Huntiella moniliformis* multi-modular NRPS cluster_Contig 10 (JMSH00000010) | | | | |  |  |  |
| --- | --- | --- | --- | --- | --- | --- | --- |
| Gene name | Size (aa) | Location on the contig | Species | E value | % Coverage | % identity | Accession number of top blast hit |
| Hydroxymate type ferrichrome siderophore peptide synthase | 4958 | 384688-400320 | *Scedosporium apiospermum* | 0 | 96 | 40 | XP_016639834 |
| L-ornithine N-5 monooxygenase | 528 | 374979-376635 | *Fusarium oxysporum* | 0 | 99 | 54 | PCD43854 |
| Endothiapepsin | 413 | 373044-374285 | *Nectria ditissima* | 1e-163 | 100 | 57 | KPM38239 |
| Hypothetical | 152 | 369599-370160 | *Scedosporium apiospermum* | 1e-17 | 88 | 36 | XP_016639846 |
| Hypothetical | 283 | 363872-364723 | *Pseudogymnoascus* sp | 4e-17 | 94 | 33 | OBT55546 |
| Mediator of RNA polymerase-II transcription subutit 7 | 256 | 362779-363604 | *Nectria haematococca* | 2e-82 | 95 | 52 | XP_003044025 |
| Membrane protein | 1002 | 359094-362638 | *Colletotrichum tofieldiae* | 0 | 75 | 56 | KZL73724 |
| Hydrolase | 250 | 357903-358753 | *Fusarium avenaceum* | 5e-98 | 100 | 55 | KIL90716 |

| M-1) *Huntiella omanensis* monomodular NRPS cluster_Contig 6521 (JSUI000006521) | | | | |  |  |  |
| --- | --- | --- | --- | --- | --- | --- | --- |
| Gene name | Size (aa) | Location on the contig | Species | E value | % Coverage | % identity | Accession number of top blast hit |
| Hypothetical | 649 | 34581-36919 | *Verticillium longisporum* | 1e-62 | 67 | 39 | CRK10802 |
| NRPS | 2081 | 37136-43437 | *Colletotrichum gloeosporioides* | 0 | 90 | 54 | XP_007284335 |
| CoA transferase | 577 | 46865-48708 | *Neotectria ditissima* | 0 | 98 | 74 | KPM45703 |
| Siderophore transporter | 585 | 49586-51343 | *Trichoderma quizhouense* | 0 | 94 | 53 | OPB43222 |
| Siderophore biosynthesis | 451 | 52183-53538 | *Purpureocillium lilacinum* | 0 | 99 | 62 | OAQ83990 |
| Oxidoreductase | 498 | 53646-55494 | *Colletotrichum simmondsii* | 3e-84 | 97 | 41 | KXH33196 |
| ABC-multidrug trasporter | 1363 | 58885-63037 | *Purpureocillium lilacinum* | 0 | 98 | 57 | OAQ83993 |
| Transporter | 1243 | 63443-68668 | *Scedosporium apiospermum* | 0 | 92 | 56 | XP_016644513 |
| Oxidoreductase | 685 | 68715-71241 | *Colletotrichum gloeosporioides* | 1e-95 | 61 | 43 | KXH54021 |
| Glutamine synthase | 722 | 72220-80817 | *Colletotrichum salicis* | 0 | 98 | 72 | KXH63435 |

| M-2) *Huntiella omanensis* multi-modular NRPS cluster_Contig 6485 (JSUI000006485) | | | | |  |  |  |
| --- | --- | --- | --- | --- | --- | --- | --- |
| Gene name | Size (aa) | Location on the contig | Species | E value | % Coverage | % identity | Accession number of top blast hit |
| Hydroxymate type ferrichrome siderophore peptide synthase | 4800 | 1-15106 | *Pestalotiopsis fici* | 0 | 98 | 39 | XP_007837428 |
| L-ornithine N-5 monooxygenase | 568 | 23511-25392 | *Fusarium oxysporum* | 0 | 92 | 58 | PCD43854 |
| Endothiapepsin | 413 | 26138-27379 | *Nectria ditissima* | 5e-165 | 100 | 57 | KPM38239 |
| Hypothetical | 230 | 31312-32710 | *Scedosporium apiospermum* | 2e-16 | 83 | 32 | XP_016639846 |
| Hypothetical | 172 | 38419-38937 | *Pseudogymnoascus* sp | 3e-07 | 98 | 40 | OBT55546 |
| Mediator of RNA polymerase-II transcription subutit 7 | 323 | 39947-41054 | *Fusarium langsethiae* | 1e-40 | 94 | 43 | KPA42344 |
| Membrane protein | 1489 | 42379-47068 | *Drechmeria coniospora* | 0 | 66 | 57 | KYK61075 |
| Hydrolase | 250 | 47570-48420 | *Fusarium avenaceum* | 5e-98 | 100 | 55 | KIL90716 |

| N-1) *Huntiella savannae* monomodular NRPS cluster_Contig 3 (LCZG00000003) | | | | |  |  |  |
| --- | --- | --- | --- | --- | --- | --- | --- |
| Gene name | Size (aa) | Location on the contig | Species | E value | % Coverage | % identity | Accession number of top blast hit |
| Hypothetical | 649 | 34581-36919 | *Verticillium longisporum* | 1e-62 | 67 | 39 | CRK10802 |
| NRPS | 2081 | 37136-43437 | *Colletotrichum gloeosporioides* | 0 | 90 | 54 | XP_007284335 |
| CoA transferase | 577 | 46865-48708 | *Neotectria ditissima* | 0 | 98 | 74 | KPM45703 |
| Siderophore transporter | 585 | 49586-51343 | *Trichoderma quizhouense* | 0 | 94 | 53 | OPB43222 |
| Siderophore biosynthesis | 451 | 52183-53538 | *Purpureocillium lilacinum* | 0 | 99 | 62 | OAQ83990 |
| Oxidoreductase | 498 | 53646-55494 | *Colletotrichum simmondsii* | 3e-84 | 97 | 41 | KXH33196 |
| ABC-multidrug trasporter | 1363 | 58885-63037 | *Purpureocillium lilacinum* | 0 | 98 | 57 | OAQ83993 |
| Transporter | 1243 | 63443-68668 | *Scedosporium apiospermum* | 0 | 92 | 56 | XP_016644513 |
| Oxidoreductase | 685 | 68715-71241 | *Colletotrichum gloeosporioides* | 1e-95 | 61 | 43 | KXH54021 |
| Glutamine synthase | 722 | 72220-80817 | *Colletotrichum salicis* | 0 | 98 | 72 | KXH63435 |

| N-2) *Huntiella savannae* multi-modular NRPS cluster_Contig 52 (LCZG00000052) | | | | |  |  |  |
| --- | --- | --- | --- | --- | --- | --- | --- |
| Gene name | Size (aa) | Location on the contig | Species | E value | % Coverage | % identity | Accession number of top blast hit |
| Hydroxymate type ferrichrome siderophore peptide synthase | 4800 | 1-15106 | *Pestalotiopsis fici* | 0 | 98 | 39 | XP_007837428 |
| L-ornithine N-5 monooxygenase | 568 | 23511-25392 | *Fusarium oxysporum* | 0 | 92 | 58 | PCD43854 |
| Endothiapepsin | 413 | 26138-27379 | *Nectria ditissima* | 4e-135 | 100 | 57 | KPM38239 |
| Hypothetical | 230 | 31312-32710 | *Scedosporium apiospermum* | 2e-117 | 83 | 32 | XP_016639846 |
| Hypothetical | 172 | 38419-38937 | *Pseudogymnoascus* sp | 3e-16 | 98 | 40 | OBT55546 |
| Mediator of RNA polymerase-II transcription subutit 7 | 323 | 39947-41054 | *Fusarium langsethiae* | 1e-40 | 94 | 43 | KPA42344 |
| Membrane protein | 1489 | 42379-47068 | *Drechmeria coniospora* | 0 | 66 | 57 | KYK61075 |
| Hydrolase | 250 | 47570-48420 | *Fusarium avenaceum* | 5e-98 | 100 | 55 | KIL90716 |

| O-1) *Thielaviopsis musarum* monomodular NRPS cluster_Contig 4 (LKBB00000004) | | | | |  |  |  |
| --- | --- | --- | --- | --- | --- | --- | --- |
| Gene name | Size (aa) | Location on the contig | Species | E value | % Coverage | % identity | Accession number of top blast hit |
| Ras_GTPase | 208 | 140618-141708 | *Grosmannia clavigera* | 8e-147 | 100 | 97 | XP_014176081 |
| No similarity | 1357 | 142931-147060 | - | - | - | - | - |
| Hypothetical | 378 | 147258-148473 | *Beauveria bassiana* | 3e-46 | 97 | 35 | PMB66460 |
| Hypothetical | 367 | 149631-150925 | *Trichoderma virens* | 7e-58 | 99 | 40 | XP_013952880 |
| No similarity | 448 | 158157-159503 | - | - | - | - | - |
| NRPS | 2073 | 160222-166562 | *Colletotrichum gloeosporioides* | 0 | 92 | 54 | XP_007284335 |
| CoA transferase | 577 | 170543-172382 | *Pochinia chlamydosporia* | 0 | 98 | 76 | XP_018147655 |
| Siderophore transporter | 616 | 172932-174833 | *Trichoderma citrinoviride* | 0 | 95 | 55 | XP_024745106 |
| Siderophore biosynthesis | 451 | 175670-177084 | *Stachybotrys chartarum* | 0 | 96 | 67 | KEY64463 |
| Oxidoreductase | 419 | 177211-178571 | *Colletotrichum incanum* | 3e-148 | 96 | 55 | OHW97157 |
| ABC-multidrug trasporter | 1352 | 180747-184872 | *Scedosporium apiospermum* | 0 | 98 | 58 | XP_016644514 |
| Transporter | 839 | 186014-188661 | *Colletotrichum tofieldiae* | 0 | 100 | 74 | KZL78304 |
| Geranylgeranyl transferase | 322 | 189812-190917 | *Colletotrichum incanum* | 3e-103 | 79 | 57 | KZL75621 |

| O-2) *Thielaviopsis musarum* multi-modular NRPS cluster_Contig 131 (LKBB00000131) | | | | |  |  |  |
| --- | --- | --- | --- | --- | --- | --- | --- |
| Gene name | Size (aa) | Location on the contig | Species | E value | % Coverage | % identity | Accession number of top blast hit |
| Hydroxymate type ferrichrome siderophore peptide synthase | 4810 | 4928-14284 | *Fusarium oxysporum* | 0 | 99 | 39 | EWZ42025 |
| No similarity | 103 | 14427-14781 | - | - | - | - | - |
| No similarity | 1604 | 14895-19992 | - | - | - | - | - |
| L-ornithine N-5 monooxygenase | 509 | 23912-25208 | *Fusarium poae* | 0 | 92 | 61 | OBS21156 |
| Endothiapepsin | 419 | 25567-26826 | *Nectria ditissima* | 1e-178 | 99 | 61 | KPM38239 |
| Mediator of RNA polymerase-II transcription subutit 7 | 255 | 3119-31936 | *Fusarium fujikuroi* | 2e-82 | 97 | 54 | SCO37772. |
| Hypothetical | 337 | 32402-33415 | *Colletotrichum nymphaeae* | 4e-60 | 92 | 45 | KXH63278 |

| P-1) *Thielaviopsis punctulata* monomodular NRPS cluster_Contig 782 (LAEV00000782) | | | | |  |  |  |
| --- | --- | --- | --- | --- | --- | --- | --- |
| Gene name | Size (aa) | Location on the contig | Species | E value | % Coverage | % identity | Accession number of top blast hit |
| Ras_GTPase | 208 | 77436-78556 | *Grosmannia clavigera* | 8e-147 | 100 | 97 | XP_014176081 |
| No similarity | 1342 | 72076-76315 | - | - | - | - | - |
| Hypothetical | 388 | 70614-71872 | *Beauveria bassiana* | 3e-46 | 97 | 35 | PMB66460 |
| Hypothetical | 367 | 68126-69353 | *Trichoderma virens* | 6e-59 | 98 | 40 | XP_013952880 |
| No similarity | 243 | 66653-67585 | - | - | - | - | - |
| No similarity | 612 | 59372-61333 | - | - | - | - | - |
| NRPS | 2066 | 52495-58885 | *Colletotrichum gloeosporioides* | 0 | 86 | 57 | XP_007284335 |
| CoA transferase | 574 | 46729-48552 | *Pochinia chlamydosporia* | 0 | 98 | 76 | XP_018147655 |
| Siderophore transporter | 558 | 44480-46156 | *Trichoderma citrinoviride* | 0 | 91 | 58 | XP_024745106 |
| Siderophore biosynthesis | 451 | 42077-43486 | *Purpureocillium lilacinum* | 0 | 99 | 64 | OAQ83990 |
| Oxidoreductase | 419 | 40667-41977 | *Colletotrichum incanum* | 1e-150 | 96 | 56 | OHW97157 |
| ABC-multidrug trasporter | 1374 | 34218-38342 | *Scedosporium apiospermum* | 0 | 96 | 58 | XP_016644514 |
| Transporter | 1233 | 28225-33041 | *Colletotrichum higginsianum* | 0 | 67 | 73 | XP_018152026 |

| P-2) *Thielaviopsis punctulata* multi-modular NRPS cluster_Contig 405 (LAEV00000405) | | | | |  |  |  |
| --- | --- | --- | --- | --- | --- | --- | --- |
| Gene name | Size (aa) | Location on the contig | Species | E value | % Coverage | % identity | Accession number of top blast hit |
| Hydroxymate type ferrichrome siderophore peptide synthase | 4697 | 44375-58733 | *Scedosporium apiospermum* | 0 | 99 | 41 | XP_016639834 |
| L-ornithine N-5 monooxygenase | 516 | 38397-40068 | *Fusarium avenaceum* | 0 | 92 | 60 | KIL93640 |
| Endothiapepsin | 418 | 36850-38106 | *Nectria ditissima* | 8e-175 | 99 | 60 | KPM38239 |
| Hypothetical | 348 | 30987-32033 | *Fusarium venenatum* | 7e-54 | 99 | 43 | CEI68809 |
| F-box domain | 339 | 29339-30675 | *Ophiocordyceps unilateralis* |  |  |  | PFH60124 |

| Q-1) *Endoconidiophora laricicola* monomodular NRPS cluster_Contig 63 (LXGT00000063) | | | | |  |  |  |
| --- | --- | --- | --- | --- | --- | --- | --- |
| Gene name | Size (aa) | Location on the contig | Species | E value | % Coverage | % identity | Accession number of top blast hit |
| Transporter | 843 | 55938-58665 | *Colletotrichium incanum* | 0 | 100 | 73 | OHW98426 |
| Leptomycin B resistance protein | 1368 | 60436-64603 | *Purpureocillium lilacinum* | 0 | 97 | 58 | OAQ83393 |
| Oxoreductase | 418 | 69286-70828 | *Trichoderma gamsii* | 1e-155 | 97 | 53 | PNP37940 |
| Siderophore biosynthesis protein | 452 | 71094-72532 | *Prechmeria coniospora* | 0 | 99 | 63 | KYK58456 |
| Acyl-CoA synthetase | 576 | 75486-77380 | *Neonectria ditissima* | 0 | 98 | 75 | KPM45703 |
| NRPS | 2075 | 82078-88480 | *Theilaviopsis punctulata* | 0 | 100 | 76 | KKA29546 |
| Hypothetical | 658 | 90388-92589 | *Lomentospora prolificans* | 2e-81 | 67 | 36 | PKS12262 |
| Hypothetical | 364 | 102529-103761 | *Trichoderma hazianum* | 99 | 0 | 64 | OAQ83990 |

| Q-2) *Endoconidiophora laricicola* multi-modular NRPS cluster_Contig 376 (LXGT00000376) | | | | |  |  |  |
| --- | --- | --- | --- | --- | --- | --- | --- |
| Gene name | Size (aa) | Location on the contig | Species | E value | % Coverage | % identity | Accession number of top blast hit |
| Glutathione transferase | 232 | 664-1427 | *Umbilicaria pustulata* | 3e-26 | 86 | 37 | SLM39268 |
| Transposase | 333 | 6275-7575 | *Fusarium oxysporum* | 7e-133 | 88 | 70 | ENH66637 |
| Hydroxymate type ferrichrome siderophore peptide synthase | 4810 | 8168-22785 | *Scedosporium apiospermum* | 0 | 99 | 40 | XP_016639834 |
| L-ornithine N-5 monooxygenase | 523 | 32170-33814 | *Fusarium avenaceum* | 0 | 91 | 59 | KIL93640 |
| Endothiapepsin | 415 | 35185-36432 | *Nectria ditissima* | 0 | 100 | 62 | KPM38239 |
| Mediator of RNA polymerase-II transcription subutit 7 | 297 | 39445-40399 | *Fusarium poae* | 3e-77 | 83 | 51 | OBS21164 |
| Hypothetical | 323 | 40811-41782 | *Fusarium langsethiae* | 9e-28 | 82 | 41 | KPA42345 |

| R-1) *Endoconidiophora polonica* monomodular NRPS cluster_Contig 369 (LXKZ00000369) | | | | |  |  |  |
| --- | --- | --- | --- | --- | --- | --- | --- |
| Gene name | Size (aa) | Location on the contig | Species | E value | % Coverage | % identity | Accession number of top blast hit |
| Ras_GTPase | 229 | 65181-66538 | *Grosmannia clavigera* | 6e-141 | 100 | 89 | XP_014176081 |
| Hypothetical | 357 | 54635-55845 | *Stachybotrys chlorohalonata* | 7e-56 | 92 | 41 | KFA61745 |
| Hypothetical | 658 | 42479-45109 | *Diaporthe ampelina* | 8e-31 | 50 | 31 | PKS12262 |
| NRPS | 2074 | 34176-40574 | *Colletotrichum gloeosporioides* | 0 | 86 | 56 | XP_007284335 |
| CoA transferase | 584 | 27375-29267 | *Neotectria ditissima* | 0 | 98 | 74 | KPM45703 |
| Siderophore biosynthesis | 452 | 23021-24452 | *Purpureocillium lilacinum* | 0 | 98 | 64 | OAQ83990 |
| Oxidoreductase | 418 | 21209-22750 | *Trichoderma gamsii* | 5e-156 | 98 | 53 | PNP37940 |
| ABC-multidrug trasporter | 1353 | 12586-16753 | *Purpureocillium lilacinum* | 0 | 97 | 57 | OAQ83993 |
| Transporter | 843 | 8222-10949 | *Colletotrichum incanum* | 0 | 100 | 74 | OHW98426 |
| PH domain | 1498 | 3239-7791 | *Colletotrichum chlorophyti* | 0 | 96 | 48 | KXH54021 |

| R-2) *Endoconidiophora polonica* multi-modular NRPS cluster_Contig 625 (LXKZ00000625) | | | | |  |  |  |
| --- | --- | --- | --- | --- | --- | --- | --- |
| Gene name | Size (aa) | Location on the contig | Species | E value | % Coverage | % identity | Accession number of top blast hit |
| Hydroxymate type ferrichrome siderophore peptide synthase | 4939 | 1-15495 | *Scedosporium apiospermum* | 0 | 99 | 40 | XP_016639834 |
| L-ornithine N-5 monooxygenase | 520 | 28287-29935 | *Fusarium longsethiae* | 0 | 99 | 57 | KPA42335 |
| Endothiapepsin | 420 | 30734-31996 | *Nectria ditissima* | 8e-161 | 99 | 57 | KPM38239 |
| Mediator of RNA polymerase-II transcription subutit 7 | 253 | 36085--36931 | *Fusarium graminearum* | 3e-89 | 96 | 56 | XP_011323885 |
| Hypothetical | 360 | 37350-38432 | *Fusarium langsethiae* | 9e-28 | 82 | 41 | KPA42345 |

| S-1) *Davidsoniella virescens* monomodular NRPS cluster_Contig 219 (LJZU000000219) | | | | |  |  |  |
| --- | --- | --- | --- | --- | --- | --- | --- |
| Gene name | Size (aa) | Location on the contig | Species | E value | % Coverage | % identity | Accession number of top blast hit |
| NRPS | 2058 | 6581-12991 | *Thelaviopsis punctulata* | 0 | 100 | 79 | KKA29546 |
| Acyl-CoA Synthetase | 589 | 20537-22468 | *Neonectria ditissima* | 0 | 98 | 73 | KPM45703 |
| Siderophore biosynthetic protein | 452 | 27550-28992 | *Colletotrichium hymphaeae* | 0 | 96 | 64 | KXH42363 |
| Oxidoreductase | 427 | 29213-30785 | *Nectria haematococca* | 2e-151 | 96 | 56 | XP_003048494 |
| Leptomycin B resistance protein | 1367 | 35507-39671 | *Purpureocillium lilacinum* | 0 | 98 | 58 | OAQ83993 |
| SPX domain | 1030 | 40078-43976 | *Colletotrichium gloesporioides* | 0 | 85 | 73 | ELA37746 |
| PH domain | 1497 | 44403-48952 | *Colletotricium chlorophyti* | 0 | 96 | 47 | OLN81630 |

| S-2) *Davidsoniella virescens* multi-modular NRPS cluster Contig 225 (LJZU000000225) | | | | |  |  |  |
| --- | --- | --- | --- | --- | --- | --- | --- |
| Gene name | Size (aa) | Location on the contig | Species | E value | % Coverage | % identity | Accession number of top blast hit |
| F-box domain | 194 | 68003-68824 | *Colletotrichium graminicila* | 2e-77 | 97 | 61 | XP_008095400 |
| Hypothetical | 353 | 69398-70459 | *Fusarium guenaceum* | 2e-54 | 98 | 40 | KIL93607 |
| Transcription subunit 7 | 254 | 70871-71731 | *Colletotrichium salicis* | 2e-85 | 97 | 53 | KXH53359 |
| Peptidase A1 | 423 | 75705-76976 | *Neonectria ditissima* | 6e-160 | 100 | 57 | KPM38239 |
| L-ornithine N-5-monooxygenase | 528 | 77871-79530 | *Fusarium pseudograminearum* | 0 | 94 | 58 | XP_009260674 |
| NRPS | 4968 | 90563-105723 | *Scedosporium apiospermum* | 0 | 99 | 40 | XP_016639834 |
| HD Domain | 251 | 106520-107548 | *Colletotrichium chlorophyti* | 5e-89 | 92 | 57 | OLN87347 |
| Hypothetical | 143 | 109001-109486 | *Scedosporum apiospermum* | 3e-57 | 97 | 57 | XP_016640357 |
| Transcriptional adapter 2 | 538 | 111648-113348 | *Colletotrichium sublineola* | 0 | 100 | 74 | KDN70890 |
| Galactose-1-phosphate uridylyltransferase | 386 | 114287-115699 | *Lomentospora prolificans* | 0 | 98 | 68 | PKS07387 |

| V-1) *Ambrosiella xylebori* multi modular NRPS Contig 4 (PCDO01000004) | | | | |  |  |  |
| --- | --- | --- | --- | --- | --- | --- | --- |
| Gene name | Size (aa) | Location on the contig | Species | E value | % Coverage | % identity | Accession number of top blast hit |
| HIR 1 | 232 | 27849-31063 | *Trichoderma patareesei* | 0 | 99 | 70 | OTA00320 |
| UDP-galactose transporter | 333 | 33739-35426 | *Pseudomassariella vexata* | 0 | 95 | 61 | ORY61530 |
| Mannose-1-phosphate guanylyltransferase | 523 | 35787-37176 | *Fusarium avenaceum* | 0 | 100 | 80 | KIL93620 |
| NRPS | 4810 | 49879-64319 | *Ceratocystis fimbriata* | 0 | 99 | 39 | PHH49945 |
| L-ornithine 5-monooxygenase | 415 | 73111-74807 | *Fusarium oxysporum* | 0 | 99 | 56 | PCD43854 |
| Peptidase A1 | 297 | 76066-77298 | *Neonectria ditissima* | 6e-178 | 99 | 61 | KPM38239 |
| Hypothetical | 323 | 88612-91585 | *Neonentria ditissima* | 0 | 80 | 54 | KPM43303 |

| V-2) *Ambrosiella xylebori* mono modular NRPS Contig 1 (PCDO01000001) | | | | |  |  |  |
| --- | --- | --- | --- | --- | --- | --- | --- |
| Gene name | Size (aa) | Location on the contig | Species | E value | % Coverage | % identity | Accession number of top blast hit |
| NRPS | 2110 | 158842-165401 | *Colletotrichum orbiculare* | 0 | 86 | 55 | ENH80243 |
| CoA transferase | 577 | 171906-173804 | *Neonectria ditissima* | 0 | 98 | 74 | KPM45703 |
| Siderophore transporter | 560 | 175045-176727 | *Trichoderma citrinoviride* | 0 | 95 | 54 | XP_024745106 |
| Siderophore biosynthesis | 452 | 182043-183401 | *Purpureocillium lilacinum* | 0 | 99 | 65 | OAQ83990 |
| Oxidoreductase | 419 | 183672-185280 | *Cordyceps militaris* | 1e-146 | 96 | 55 | XP_006671902 |
| ABC-multidrug transporter | 1371 | 189996-194333 | *Metarhizium acridum* | 0 | 98 | 57 | XP_007808197 |
| Transporter | 845 | 195825-198390 | *Colletotrichum incanum* | 0 | 100 | 74 | OHW98426 |

| W-1-A) *Davidsoniella australis* mono modular NRPS contig 1 | | | | |  |  |  |
| --- | --- | --- | --- | --- | --- | --- | --- |
| Gene name | Size (aa) | Location on the contig | Species | E value | % Coverage | % identity | Accession number of top blast hit |
| Siderophore iron transporter | 208 | 845-2545 | *Chaetomium globosum* | 0 | 97 | 54 | XP_001227831 |
| Acyl-CoA Synthetase | 374 | 3702-5633 | *Neonectria ditissima* | 0 | 98 | 75 | KPM45703 |
| NRPS | 799 | 13876-20305 | *Thielaviopsis punctulata* | 0 | 100 | 80 | KKA29546 |
| Hypothetical | 562 | 38791-40007 | *Trichoderma hazianum* | 2e-57 | 97 | 41 | PNP45214 |
| RAS GTPase | 2092 | 49485-50850 | *Grosmannia clavigera* | 1e-140 | 90 | 96 | XP_014176081 |
| W-1-B) *Davidsoniella australis* mono modular NRPS contig 393 | | | | | | | |
| Gene name | Size (aa) | Location on the contig | Species | E value | % Coverage | % identity | Accession number of top blast hit |
| ABC-multidrug transporter | 1267 | 9102-11586 | *Purpureocillium lilacinum* | 0 | 97 | 57 | OAQ83993 |
| Transporter | 848 | 13425-16894 | *Purpureocillium lilacinum* | 0 | 96 | 59 | OAQ83993 |
| Oxidoreductase | 421 | 22105-23676 | *Nectria haematococca* | 2e-26 | 97 | 58 | XP_003048494 |
| Siderophore biosynthesis | 455 | 23895-24712 | *Colletotrichium hymphaeae* | 0 | 97 | 66 | KXH42363 |

| W-2) *Davidsoniella australis* multi modular NRPS contig 143 | | | | |  |  |  |
| --- | --- | --- | --- | --- | --- | --- | --- |
| Gene name | Size (aa) | Location on the contig | Species | E value | % Coverage | % identity | Accession number of top blast hit |
| Nucleoporin | 232 | 664-1427 | *Colletotrichium simmondsii* | 0 | 99 | 66 | KXH44332 |
| 3-ketoacyl-CoA reductase | 333 | 800-5192 | *Colletotrichium higginsianum* | 2e-171 | 97 | 70 | XP_018151184 |
| Ergosterol biosynthetic protein 28 | 4810 | 6309-7485 | *Colletotricium gloesporioides* | 3e-69 | 97 | 72 | EQB54482 |
| Galactose-1-phosphate uridylyltransferase | 523 | 7946-8525 | *Lomentospora prolificans* | 0 | 98 | 68 | PKS07387 |
| Transcriptional adapter 2 | 415 | 8616-10034 | *Colletotricium sublineola* | 0 | 95 | 73 | KDN70890 |
| Hypothetical | 297 | 10992-12653 | *Scedosporium apiospermum* | 5e-59 | 97 | 58 | XP_016640357 |
| HD Domain | 323 | 14724-15219 | *Hypoxylon* Spp | 6e-99 | 95 | 64 | OTB00397 |
| NRPS | 4750 | 18529-33759 | *Scedosporium apiospermum* | 0 | 100 | 39 | XP_016639834 |

| X-1) *Davidsoniella neocaledoniae* multi modular NRPS Contig 6 | | | | |  |  |  |
| --- | --- | --- | --- | --- | --- | --- | --- |
| Gene name | Size (aa) | Location on the contig | Species | E value | % Coverage | % identity | Accession number of top blast hit |
| NRPS | 208 | 10101-11196 | *Ceratocystis fimbriata* | 0 | 99 | 48 | PHH49945 |
| HD Domain | 374 | 12451-13304 | *Hypoxylon* | 1e-98 | 85 | 64 | OTB00397 |
| Hypothetical | 799 | 17267-18477 | *Scedosporium apiospermum* | 2e-58 | 97 | 58 | XP_016640357 |
| Transcriptional activator | 562 | 19710-23430 | *Colletotrichium sublinecola* | 0 | 100 | 74 | KDN70890 |
| Galactose-1-phosphate uridylyltransferase | 2092 | 29439-31379 | *Colletotrichium graminicola* | 5e-123 | 96 | 64 | XP_008100599 |
| Ergosterol biosynthetic protein 28 | 577 | 32137-38636 | *Escovopsis chlorophyte* | 3e-70 | 88 | 63 | OLN97423 |
| 3-ketoacyl-CoA Reductase | 586 | 44922-46799 | *Colletotrichium higgnsianum* | 3e-172 | 97 | 71 | XP_018151184 |
| Nucleoporin | 452 | 48845-50814 | *Colletotrichium simmondsii* | 0 | 99 | 66 | KXH44332 |
| Sugar transporter STL1 | 421 | 52470-53828 | *Verticillium dahliae* | 0 | 76 | 71 | PNH43205 |

| X-2-A) *Davidsoniella neocaledoniae* Mono modular NRPS Contig 1989 | | | | |  |  |  |
| --- | --- | --- | --- | --- | --- | --- | --- |
| Gene name | Size (aa) | Location on the contig | Species | E value | % Coverage | % identity | Accession number of top blast hit |
| Siderophore iron transporter | 208 | 845-2545 | *Chaetomium globosum* | 0 | 97 | 54 | XP_001227831 |
| Acyl-CoA Synthetase | 374 | 3702-5633 | *Neonectria ditissima* | 0 | 98 | 75 | KPM45703 |
| NRPS | 799 | 13876-20305 | *Thielaviopsis punctulata* | 0 | 100 | 80 | KKA29546 |
| Hypothetical | 562 | 38791-40007 | *Trichoderma hazianum* | 2e-57 | 97 | 41 | PNP45214 |
| RAS GTPase | 2092 | 49485-50850 | *Grosmannia clavigera* | 1e-140 | 90 | 96 | XP_014176081 |
| X-2-B) *Davidsoniella neocaledoniae* Mono modular NRPS Contig 280 | | | | | | | |
| Gene name | Size (aa) | Location on the contig | Species | E value | % Coverage | % identity | Accession number of top blast hit |
| ABC-multidrug transporter | 1264 | 3235-7431 | *Purpureocillium lilacinum* | 0 | 98 | 57 | OAQ83993 |
| Transporter | 845 | 8475-11637 | *Purpureocillium lilacinum* | 0 | 96 | 59 | OAQ83993 |
| X-2-C) *Davidsoniella neocaledoniae* Mono modular NRPS Contig 3038 | | | | |  |  |  |
| Gene name | Size (aa) | Location on the contig | Species | E value | % Coverage | % identity | Accession number of top blast hit |
| Oxidoreductase | 397 | 1-1112 | *Nectria haematococca* | 1e-154 | 98 | 59 | XP_003048494 |
| Siderophore biosynthesis | 456 | 1135-2589 | *Colletotrichium hymphaeae* | 0 | 96 | 66 | KXH42363 |

| W-1) *Berkeleyomyces basicola* monomodular NRPS cluster_Contig8 | | | | |  |  |  |
| --- | --- | --- | --- | --- | --- | --- | --- |
| Gene name | Size (aa) | Location on the contig | Species | E value | % Coverage | % identity | Accession number of top blast hit |
| GTPase | 575 | 1671203-1674202 | *Grosmannia clavigera* | 4e-139 | 35 | 97 | XP_014176081 |
| Hypothetical (PAPA) | 319 | 1674702-1675822 | *Metarhizium rileyi* | 2e-60 | 98 | 45 | OAA44328 |
| Hypothetical | 666 | 1680570-1682570 | *Colletotrichium gloeosporioides* | 2e-74 | 67 | 39 | EQB54599 |
| NRPS | 2098 | 1683637-1675822 | *Colletotrichium gloeosporioides* | 0 | 86 | 54 | EQB69356 |
| Acyl-CoA Synthetase | 579 | 1692143-1694003 | *Trichoderma citrinoviridae* | 0 | 97 | 76 | XP_024745104 |
| Siderophore iron transposase | 570 | 1694626-1696507 | *Thermothelomyces thermophile* | 0 | 89 | 57 | XP_003665004 |
| Siderophore biosynthesis protein | 448 | 1697003-1698433 | *Metarhizium brunneum* | 0 | 98 | 64 | XP_014549676 |
| ABC transporter | 2302 | 1701212-1709524 | *Pochonia chlamyoporia* | 0 | 98 | 60 | XP_018147652 |

| W-2) *Berkeleyomyces basicola* multi-modular NRPS cluster_Contig 12 | | | | |  |  |  |
| --- | --- | --- | --- | --- | --- | --- | --- |
| Gene name | Size (aa) | Location on the contig | Species | E value | % Coverage | % identity | Accession number of top blast hit |
| Hypothetical | 371 | 1-1493 | *Fusarium oxysporum* | 0 | 100 | 69 | EXL64588 |
| NRPS | 4885 | 6645-21513 |  |  |  |  |  |
| L-ornithine 5-monooxygenase | 531 | 27720-29384 | *Fusarium oxysporum* | 0 | 99 | 56 | PCD43854 |
| RNA polymerase II subunit 7 | 259 | 31358-32199 | *Colletotrichium simmondsii* | 5e-87 | 97 | 53 | KXH42963 |
| Hypothetical | 165 | 43568-44221 | *Colletotrichium incanum* | 4e-42 | 99 | 52 | OHW92273 |
| Membrane protein | 1056 | 45197-48475 | *Colletotrichium incanum* | 0 | 82 | 57 | KZL86520 |
| Transcription factor | 790 | 49391-51907 | *Lomentospora prolificans* | 0 | 99 | 47 | PKS11306 |
